# Structural diversity of halocarbonyl molybdenum and tungsten PNP pincer complexes through ligand modifications†
†Electronic supplementary information (ESI) available: Complete crystallographic data and technical details in CIF format for **5a**, **5b**·1.5CH_2_Cl_2_, **6a**·CDCl_3_, **7a**, **8a** and **10**. Atomic coordinates for all DFT optimized structures. CCDC 1478552–1478557. For ESI and crystallographic data in CIF or other electronic format see DOI: 10.1039/c6dt02251k



**DOI:** 10.1039/c6dt02251k

**Published:** 2016-07-20

**Authors:** Sara R. M. M. de Aguiar, Berthold Stöger, Ernst Pittenauer, Günter Allmaier, Luis F. Veiros, Karl Kirchner

**Affiliations:** a Institute of Applied Synthetic Chemistry , Vienna University of Technology , Getreidemarkt 9 , A-1060 Vienna , Austria . Email: kkirch@mail.tuwien.ac.at; b Institute of Chemical Technologies and Analytics , Vienna University of Technology , Getreidemarkt 9 , A-1060 Vienna , Austria; c Centro de Química Estrutural , Instituto Superior Técnico , Universidade de Lisboa , Av. Rovisco Pais No. 1 , 1049-001 Lisboa , Portugal

## Abstract

This work presents a comparative study of a series of halocarbonyl Mo(ii) and W(ii) complexes of the types [M(PNP)(CO)_3_X]X and [M(PNP)(CO)_2_X_2_] (M = Mo, W; X = I, Br), featuring PNP pincer ligands based on a 2,6-diaminopyridine scaffold. The complexes were prepared and fully characterized. The syntheses of these complexes were accomplished by treatment of [M(PNP)(CO)_3_] with stoichiometric amounts of I_2_ and Br_2_, respectively. The modification of the 2,6-diaminopyridine scaffold by introducing NMe and NPh instead of NH spacers with concomitant modification of the phosphine moieties changed the steric and electronic properties of the PNP ligand significantly. While in the case of NH linkers exclusively cationic seven-coordinate complexes of the type [M(PNP)(CO)_3_X]^+^ were obtained with NMe and NPh spacers neutral seven-coordinate complexes of the type [M(PNP)(CO)_2_X_2_] were afforded. In the case of the latter, when the reaction is performed in the presence of CO also [M(PNP)(CO)_3_X]^+^ complexes are formed which slowly lose CO to give [M(PNP)(CO)_2_X_2_]. The halocarbonyl tungsten chemistry parallels that of molybdenum. The only exception is molybdenum in conjunction with the PNP^Me^-iPr ligand, where the coordinatively unsaturated complex [Mo(PNP^Me^-iPr)(CO)X_2_] is formed. DFT mechanistic studies reveal that the seven-coordinate complexes should be the thermodynamic as well as the kinetic products. Since [Mo(PNP^Me^-iPr)(CO)X_2_] is the observed product it suggests that the reaction follows an alternative path. Structures of representative complexes were determined by X-ray single crystal analyses.

## Abstract 2

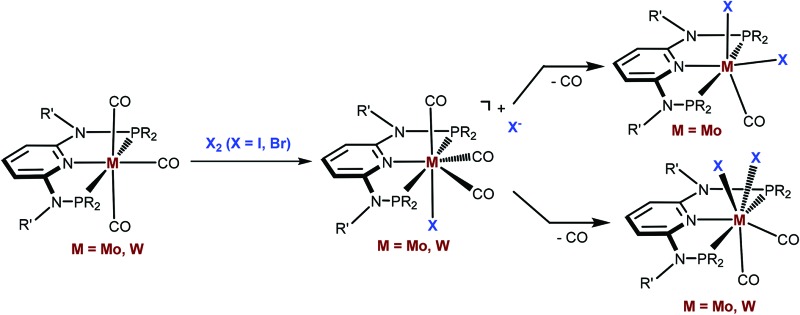
A comparative study of a series of halocarbonyl Mo(ii) and W(ii) complexes featuring PNP pincer ligands based on a 2,6-diaminopyridine scaffold is presented.

## Introduction

**Scheme 1:**
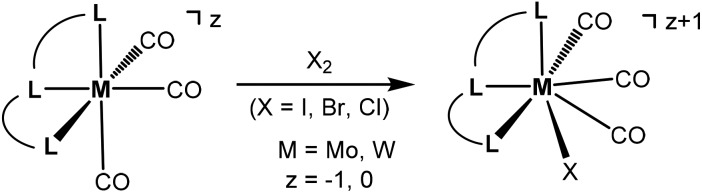
Oxidative addition of X_2_ to *fac*-[ML_3_(CO)_3_]^z^.

**Scheme 2:**
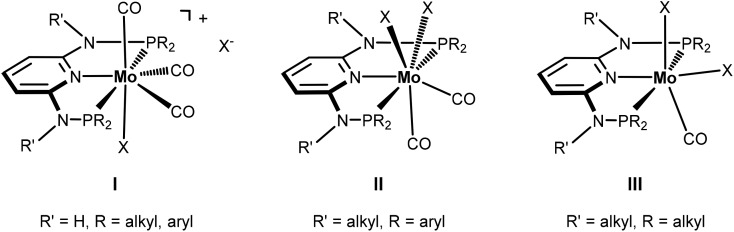
Structural diversity of halocarbonyl Mo PNP pincer complexes.

Seven coordinate halocarbonyl Mo(ii) and W(ii) complexes of the type [ML_3_(CO)_3_X] where M is Mo, W; X = Cl, Br, I and L_3_ are neutral (*z* = 0) or anionic (*z* = –1) tridentate ligands adopting *fac* geometries such as Cp, Cp*, trispyrazolylborates, trispyrazolylmethanes, 1,4,7-triazacyclononane, or 1,5,9-triphosphacyclododecane are a common class of compounds. These are typically formed *via* oxidative addition of X_2_ to M(0) complexes *fac*-[ML_3_(CO)_3_]^*z*^ as shown in Scheme 1.^1^ As L_3_ ligands with a *mer* geometry are concerned such reactions have been rarely studied. Templeton and coworkers described the synthesis of a series of halocarbonyl tungsten pincer complexes featuring the silazane-based PNP pincer-type ligand HN(SiMe_2_CH_2_PPh_2_)_2_.^2^ We have recently described the synthesis of a series of Mo(ii) PNP halocarbonyl complexes of the type [Mo(PNP)(CO)_3_X]^+^ (X = I, Br, Cl) (**I**), but also complexes of the less common type [Mo(PNP)(CO)_2_X_2_] (X = I, Br, Cl, F) (**II**) and, in one case, of the unusual type [Mo(PNP)(CO)X_2_] (X = I, Br, Cl) (**III**) as illustrated in Scheme 2.^3–6^ The latter is a coordinatively unsaturated 16e low spin complex and, based on DFT calculations, is surprisingly thermodynamically unfavorable as compared to types **I** and **II** (*vide infra*). All these complexes contain PNP pincer ligands^7^ based on the 2,6-diaminopyridine scaffold where the aromatic pyridine ring and the phosphine moieties are connected *via* NH, *N*-alkyl, or *N*-aryl spacers. The latter has a profound impact on the sterics of the ligands and consequently also on the stability and reactivity of these metal complexes. Thus far, all complexes with PNP ligands bearing NH moieties, independent of the substituents at the phosphine donor, were found to form cationic seven-coordinated species of the type **I**, whereas those with PNP ligands bearing NR′ moieties (R′ ≠ H) lead to the formation of neutral dihalo species that are seven-coordinate (type **II**) or in one case with R = iPr and R′ = Me a six-coordinate complex (type **III**).

**Chart 1:**
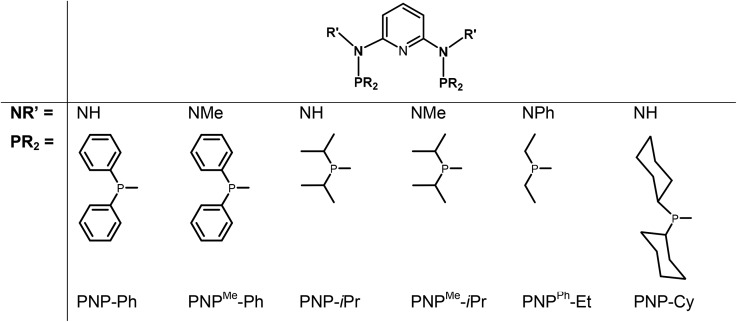
PNP ligands used for this study.

In continuation of our studies on group six PNP pincer complexes, we report here on the synthesis, characterization, and reactivity of various new halocarbonyl W(ii) as well as halocarbonyl Mo(ii) PNP pincer complexes featuring different substituents at the phosphine moieties and on the amine linkers (Chart 1). The objective is to understand the reason for the structural diversity and, in particular, whether or not halocarbonyl tungsten chemistry parallels that of molybdenum. Mechanistic aspects of the formation of halocarbonyl Mo(ii) PNP pincer complexes based on DFT calculations will be presented. X-ray structures of representative complexes will be given.

## Results and discussion

**Scheme 3:**
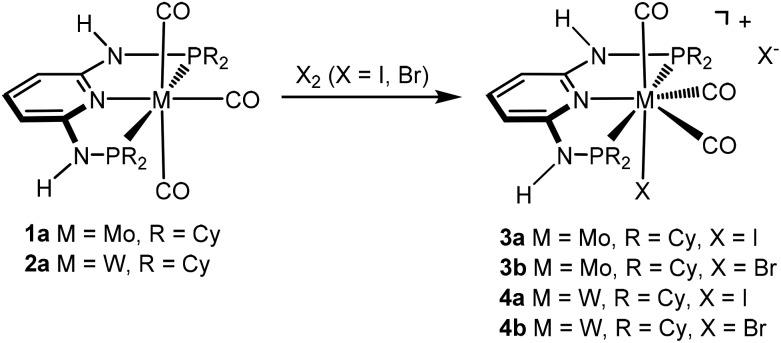
Synthesis of cationic seven-coordinate halocarbonyl molybdenum and tungsten complexes.

We have recently shown^3,4^ that the reactions of [M(PNP)(CO)_3_] (M = Mo, W) with PNP ligands featuring NH linkers exclusively gave complexes of type [M(PNP)(CO)_3_X]^+^ independent of the phosphine, *i.e.*, PPh_2_ and PiPr_2_. Here we investigate the impact of the slightly bulkier PNP-Cy ligand on the outcome of these reactions and reacted [M(PNP-Cy)(CO)_3_] (**1a**, **2a**) with stoichiometric amounts of X_2_ (X = I, Br) in CH_2_Cl_2_ at room temperature. However, again in all cases cationic seven coordinate complexes [M(PNP-Cy)(CO)_3_X]^+^ (**3**, **4**) were afforded in 85–89% yield (Scheme 3). These seven-coordinate complexes are notorious for their fluxional behavior in solution,^1,8^ and as expected, the ^31^P{^1^H} NMR spectra of **3** and **4** exhibit only one resonance. In the case of **4**, the tungsten–phosphorus coupling was observed as a doublet satellite due to ^183^W, 14% abundant with *I* = 1/2 superimposed over the dominant singlet (^1^ *J*_WP_ = 173–185 Hz). The ^13^C{^1^H} NMR spectra of **3** and **4** show two low-field triplet carbonyl resonances in a 2 : 1 ratio in the range of 234–208 ppm. In the solid-state IR spectra they display the typical three strong *ν*_CO_ bands.

**Scheme 4:**
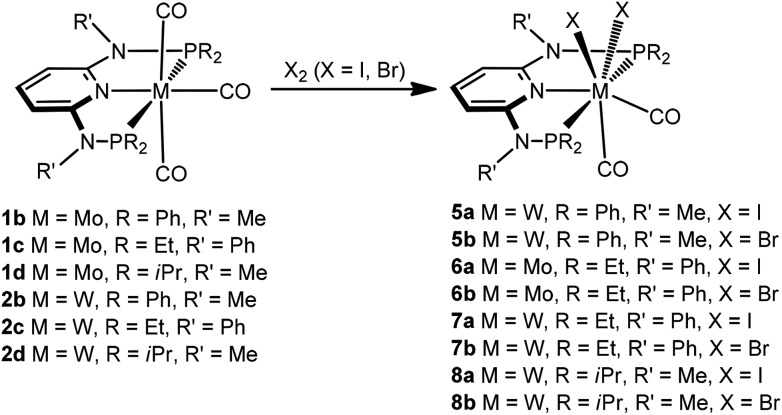
Synthesis of neutral seven-coordinate halocarbonyl molybdenum and tungsten complexes.

**Scheme 5:**
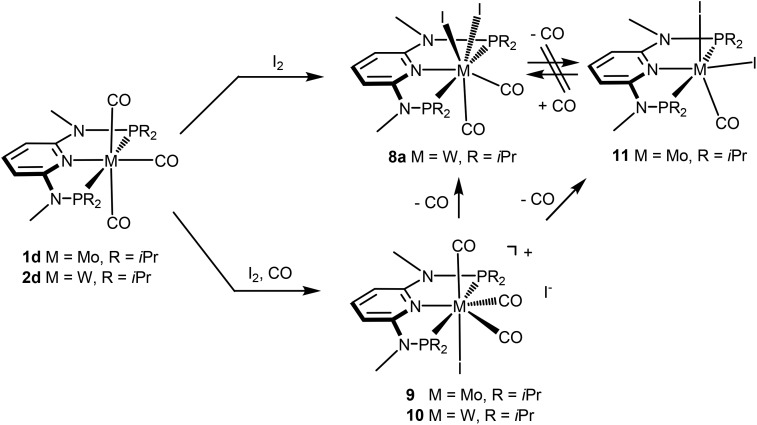
Synthesis of seven and six-coordinate iodocarbonyl molybdenum and tungsten complexes.

Next, we investigated the reactivity of [M(PNP)(CO)_3_] where the PNP ligands contain different PR_2_ and NR′ units with particular emphasis on tungsten. Treatment of **1** and **2** with stoichiometric amounts of X_2_ (X = I, Br) in CH_2_Cl_2_ at room temperature yields exclusively the neutral seven-coordinate complexes [W(PNP)(CO)_2_X_2_] (**5**, **7**, **8**) and [Mo(PNP)(CO)_2_X_2_] (**6**) in 81 to 96% isolated yields (Scheme 4). This reaction was independent of the substituents at the P and N sites, respectively, and the chemistry of tungsten strongly parallels that of molybdenum. The only exception, as reported previously,^6^ is [Mo(PNP^Me^-iPr)(CO)_3_] where instead of [Mo(PNP^Me^-iPr)(CO)_2_X_2_] the unexpected complexes [Mo(PNP^Me^-iPr)(CO)_2_X] were formed (Scheme 5, shown for X = I (**11**)).

All complexes are thermally robust red to yellow solids which are air stable in the solid state but slowly decompose in solution. Characterization was accomplished by elemental analysis and by ^1^H, ^13^C{^1^H} and ^31^P{^1^H} NMR, and IR spectroscopy. The ^13^C{^1^H} NMR spectra give rise to two characteristic low-field resonances at 225 to 263 and 204 to 238 ppm assignable to the carbonyl carbon atoms *trans* and *cis* to the pyridine nitrogen, respectively. The ^31^P{^1^H} NMR spectrum of complexes **6** gave rise to singlets at 133.3 and 138.1 ppm, respectively. The tungsten complexes **5**, **7**, and **8** exhibited singlet resonances at 108.0, 112.7, 115.4, 123.9, and 127.7 ppm with ^1^ *J*_WP_ coupling constants of 244–260 Hz. The two characteristic *ν*_CO_ frequencies are observed in the range of 1975 to 1815 cm^–1^ (*cf.* 2143 cm^–1^ in free CO) for the mutually *cis* CO ligands.

Moreover, some molybdenum and tungsten complexes were also characterized by means of ESI-MS. These studies (in positive and negative ion mode) revealed that complexes **3a**, **4a**, **5a**, and **7a** in CH_3_CN solutions in the presence of NaI remain largely intact and the peaks of the fragments of the sodiated complexes [M + Na]^+^ or iodide adducts [M + I]^–^ are intense. Further abundant fragments are [M – I]^+^ and [M – I – CO]^+^ where one iodide and CO ligand, respectively, are dissociated suggesting that one halide and one CO ligand are labile (*vide infra*).

**Fig. 1:**
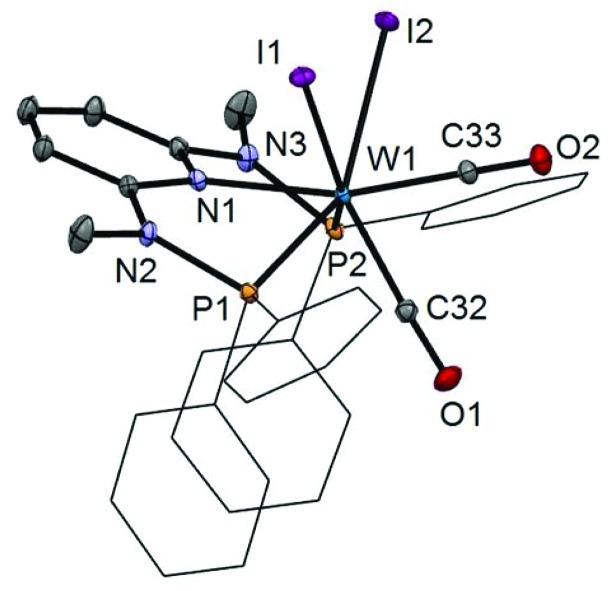
Structural view of [W(PNP^Me^-Ph)(CO)_2_I_2_] (**5a**) showing 50% thermal ellipsoids (hydrogen atoms omitted for clarity). Selected bond lengths (Å) and bond angles (°): W1–P1 2.4252(5), W1–P2, 2.4255(5), W1–N1 2.253(1), W1–C32 1.948(2), W1–C33 1.988(2), W1–I1 2.8924(3), W1–I2 2.8958(3), P1–W1–P2 113.69(1), I1–W1–I2 81.15(1), C32–W1–C33 73.46(6).

**Fig. 2:**
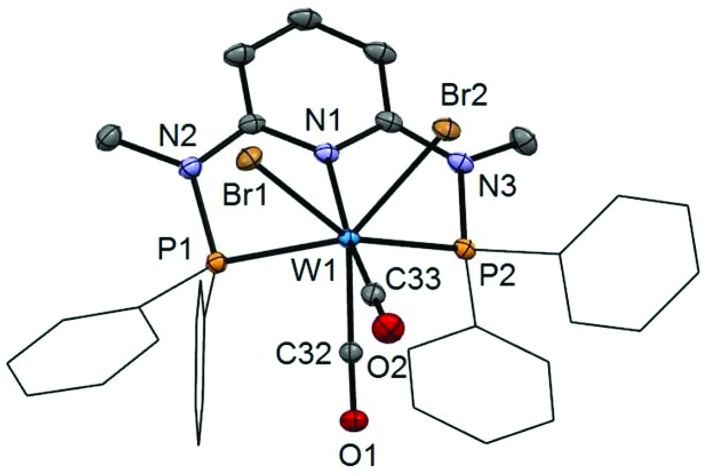
Structural view of [W(PNP^Me^-Ph)(CO)_2_Br_2_]·1.5CH_2_Cl_2_ (**5b**·1.5CH_2_Cl_2_) showing 50% thermal ellipsoids (hydrogen atoms and solvent omitted for clarity). Selected bond lengths (Å) and bond angles (°): W1–P1 2.4190(7), W1–P2 2.4203(8), W1–N1 2.240(2), W1–C32 1.953(2), W1–C33 1.984(2), W1–Br1 2.6871(3), W1–Br2 2.6444(3), P1–W1–P2 120.44(2), Br1–W1–Br2 79.43(1), C32–W1–C33 73.44(9).

**Fig. 3:**
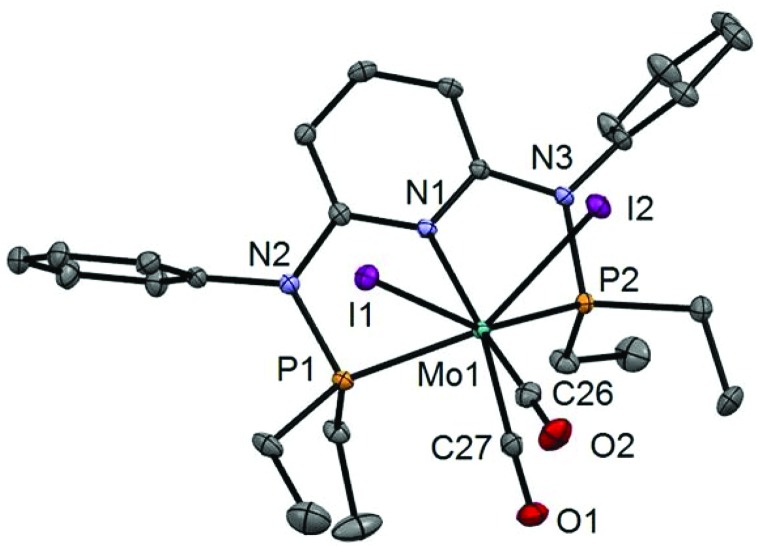
Structural view of [Mo(PNP^Ph^-Et)(CO)_2_I_2_]·CDCl_3_ (**6a**·CDCl_3_) showing 50% thermal ellipsoids (hydrogen atoms and solvent omitted for clarity). Selected bond lengths (Å) and bond angles (°): Mo1–P1 2.4384(8), Mo1–P2 2.4385(7), Mo1–C26 2.002(2), Mo1–C27 1.922(2), Mo1–I1 2.9159(5), Mo1–I2 2.9131(5), P1–Mo1–P2 111.98(2), I1–Mo1–I2 82.89(1), C26–Mo1–C27 72.01(10).

**Fig. 4:**
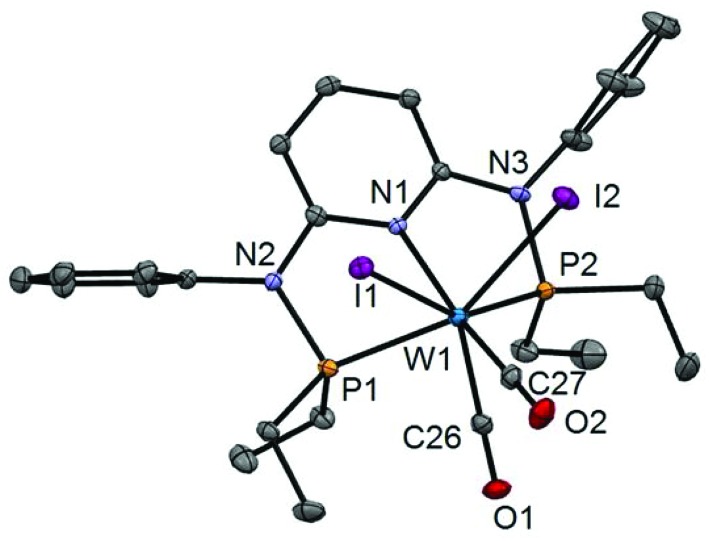
Structural view of [W(PNP^Ph^-Et)(CO)_2_I_2_] (**7a**) showing 50% thermal ellipsoids (hydrogen atoms omitted for clarity). Selected bond lengths (Å) and bond angles (°): W1–P1 2.4237(8), W1–P2 2.4322(8), W1–C26 1.932(3), W1–C27 1.997(3), W1–I1 2.9013(3), W1–I2 2.8978(3), P1–W1–P2 112.93(3), I1–W1–I2 81.40(1), C26–W1–C27 72.45(12).

**Fig. 5:**
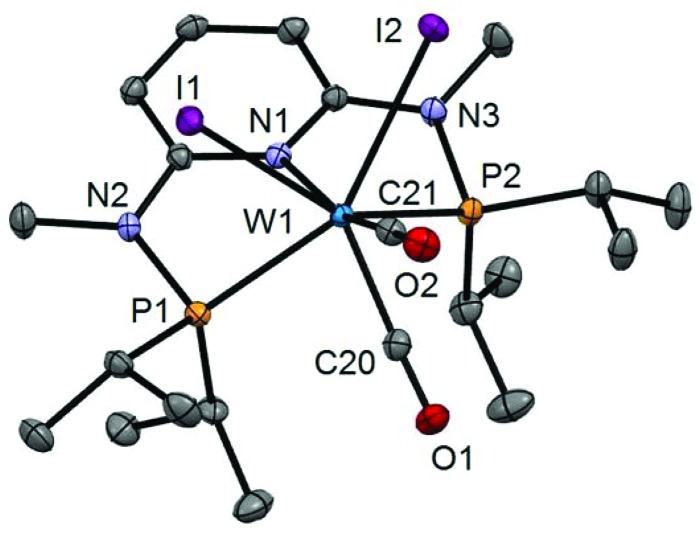
Structural view of [W(PNP^Me^-iPr)(CO)_2_I_2_] (**8a**) showing 50% thermal ellipsoids (hydrogen atoms omitted for clarity). Selected bond lengths (Å) and bond angles (°): W1–I1 2.9255(2), W1–I2 2.8999(2), W1–N1 2.2553(18), W1–P1 2.4616(7), W1–P2 2.4594(6), W1–C20 1.940(3), W1–C21 1.982(2), I1–W1–I2 79.855(5), P1–W1–P2 114.97(2), C20–W1–C21 69.54(10).

In addition to the spectroscopic characterization, the solid state structures of **5a**, **5b**, **6a**, **7a**, and **8a** were determined by single-crystal X-ray diffraction. Structural diagrams are depicted in Fig. 1–5 with selected bond distances given in the captions. The coordination geometry around the molybdenum and tungsten centers may be described as a trigonal mono capped antiprism with one CO as the capping ligand. The crystal structures show the tridentate PNP ligand bound meridionally with two carbonyl and two halide ligands filling the remaining four sites. The metal–CO bond lengths in the three complexes average to 1.96 Å (1.92–2.00 Å). The P1–M–P2 angles in the diiodo complexes **5a**, **6a**, **7a**, and **8a** are 113.69(1), 111.98(2), 112.93(3), 114.97(2)°, respectively, being significantly smaller than that in the dibromo complex **5b** where this angle is 120.44(2)°. The corresponding X–M–X and C–M–C angles in **5a**, **5b**, **6a**, **7a**, and **8a** are essentially independent of the nature of the metal and the halide being 81.15(1), 79.43(1), 82.89(1), 81.40(1) and 79.86(1)°, and 73.46(6), 73.44(9), 72.0(1), 72.5(1), and 69.5(1)°, respectively. In all complexes the metal center is significantly bent out of the least squares plane defined by the atoms of the pyridine ring.

**Scheme 6:**
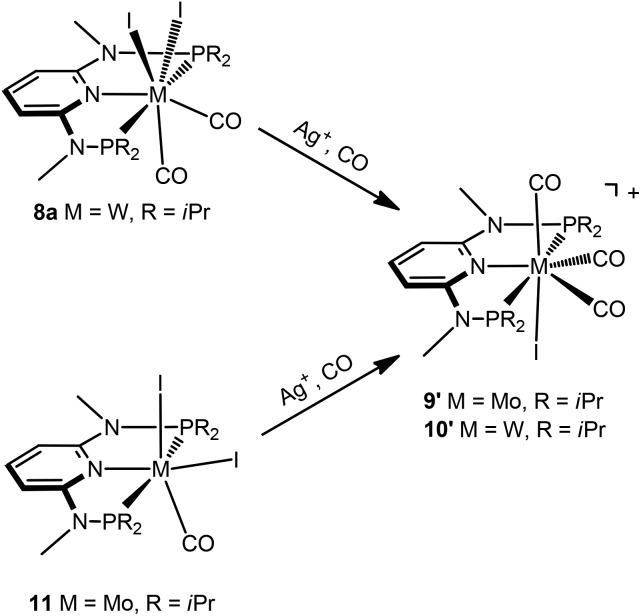
Synthesis of cationic seven coordinate iodocarbonyl molybdenum and tungsten complexes.

**Fig. 6:**
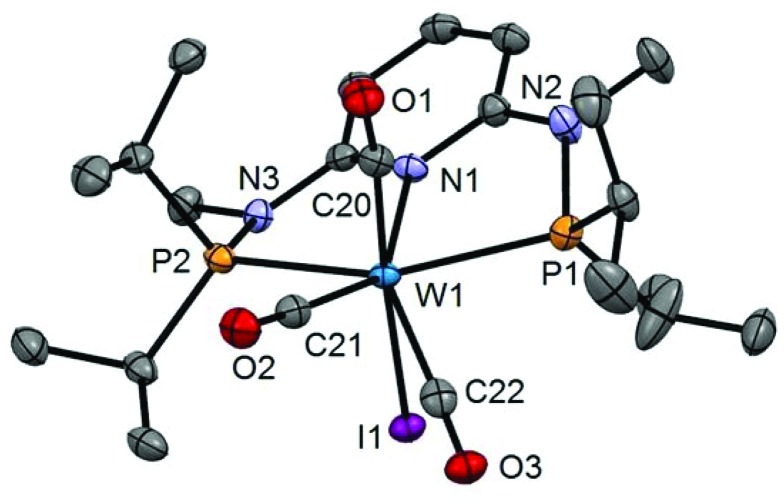
Structural view of [W(PNP^Me^-iPr)(CO)_3_I]I (**10**) showing 50% thermal ellipsoids (hydrogen atoms omitted for clarity). Selected bond lengths (Å) and bond angles (°): W1–I1 2.8835(14), W1–P1 2.5049(13), W1–P2 2.4945(17), W1–N1 2.244(3), W1–C20 1.991(3), W1–C21 1.982(4), W1–C22 2.021(3), P1–W1–P2 150.32(3).

Interestingly, when the oxidative addition of I_2_ to [Mo(PNP^Me^-iPr)(CO)_3_] (**1d**) and [W(PNP^Me^-iPr)(CO)_3_] (**2d**) was performed in the presence of CO, in both cases the seven-coordinate complexes [Mo(PNP^Me^-iPr)(CO)_3_I]I (**9**) and [W(PNP^Me^-iPr)(CO)_3_I]I (**10**) were formed (Scheme 5). These complexes could be isolated in pure form in 98 and 96% isolated yield. A structural view of complex **10** is shown in Fig. 6 with selected bond distances and angles given in the caption. However, in solution in the absence of CO, both complexes slowly release CO (about 1 h for Mo, about 3 h for W) and form again the respective neutral six- and seven-coordinate complexes [Mo(PNP^Me^-iPr)(CO)I_2_] (**11**) and [W(PNP^Me^-iPr)(CO)_2_I_2_] (**8a**). Upon treatment of **11** and **8a** with a halide scavenger (*e.g.*, AgSbF_6_) in the presence of CO, [Mo(PNP^Me^-iPr)(CO)_3_I]^+^ (**9′**) and [W(PNP^Me^-iPr)(CO)_3_I]^+^ (**10′**), respectively, are quantitatively formed again (Scheme 6). This reaction was performed in CD_2_Cl_2_ in an NMR tube and monitored by ^1^H and ^31^P{^1^H} NMR spectroscopy. Moreover, upon heating **8a** under vacuum no reaction took place and there was no evidence for the formation of [W(PNP^Me^-iPr)(CO)I_2_]. Likewise, treatment of **11** with CO for several hours did not result in the formation of [Mo(PNP^Me^-iPr)(CO)_2_I_2_]. These experiments clearly suggest that complexes of type **I** are intermediates on the way to complexes of the types **II** and **III**, but compounds of the type **II** are apparently not intermediates on the way to complexes of the type **III** (Scheme 5) .

**Scheme 7:**
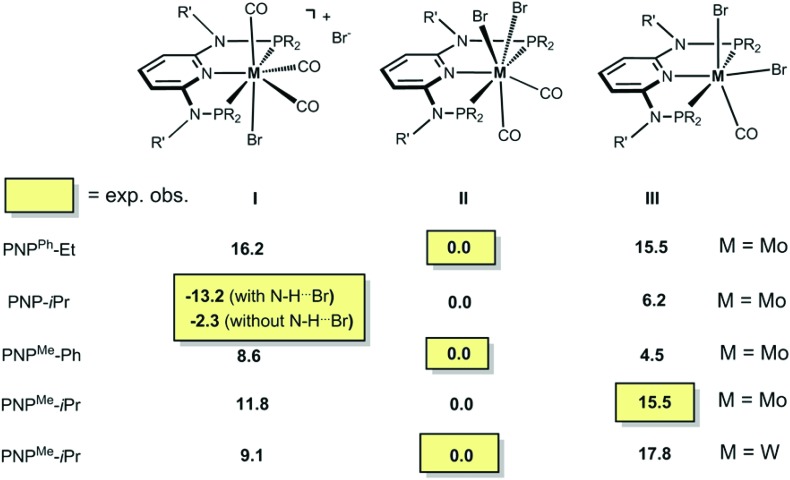
DFT calculated free energies (in kcal mol^–1^) of bromocarbonyl complexes.

In order to get a better understanding of the mechanism of the above reactions, DFT calculations were performed. First, the free energy values presented in Scheme 7 indicate that practically all reactions are under thermodynamic control. The seven-coordinate neutral complexes of type **II** are the most stable species for most combinations of the metal and PNP ligand and, thus, are the observed products in general. The cationic complex of type **I** is obtained in the case of the PNP ligand with a NH linker, in accordance with its relative stability calculated by DFT. Interestingly, the existence of an H-bond between the N–H group in the PNP ligand and the bromide counter ion stabilizes the corresponding ion pair by 10.9 kcal mol^–1^.

**Fig. 7:**
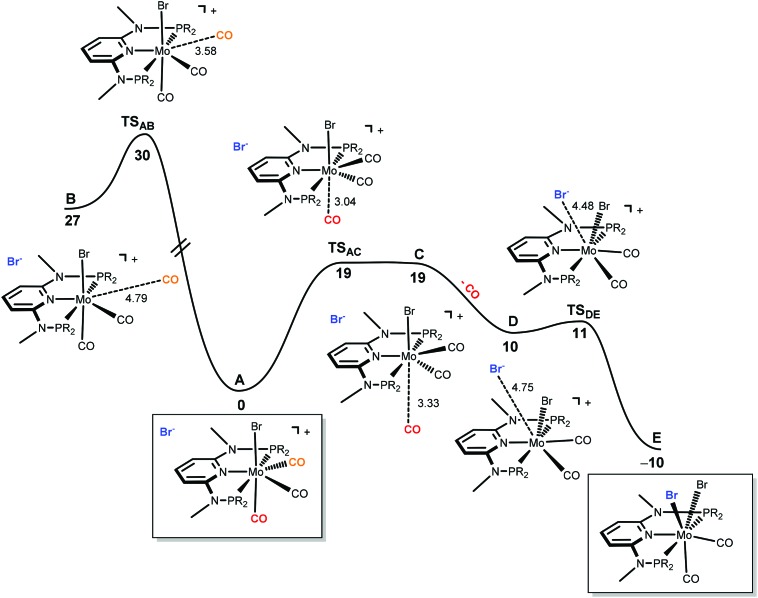
Energy profile for the dissociation of CO from [Mo(PNP^Me^-iPr)(CO)_3_Br]Br (**A**) and formation of seven-coordinate [Mo(PNP^Me^-iPr)(CO)_2_Br_2_] (**E**). The free energy values (kcal mol^–1^) are referred to **A**.

**Fig. 8:**
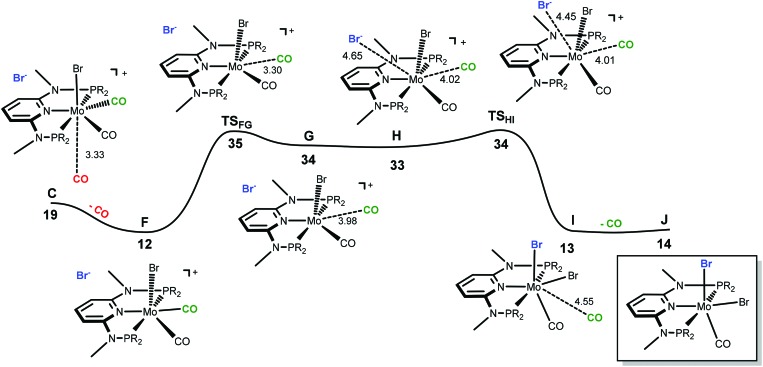
Energy profile for the formation of six-coordinate [Mo(PNP^Me^-iPr)(CO)_2_Br] (**J**). The free energy values (kcal mol^–1^) are referred to [Mo(PNP^Me^-iPr)(CO)_3_Br]Br (**A**).

The only oddity in the free values calculated for the complexes presented in Scheme 7 corresponds to the Mo complex with PNP^Me^-iPr. Here, the observed product is the six-coordinated complex with structure **III** being also the least stable one of all three species with a free energy value 15.5 kcal mol^–1^ higher than complex **II**, the most stable one. In this case the product obtained is not the thermodynamic product of the reaction and, thus, we studied the reaction mechanism trying to find a kinetic reason for the formation of complex **III**. The free energy profiles obtained are represented in Fig. 7 and 8.

The mechanism starts with the ion pair [Mo(PNP^Me^-iPr)(CO)_3_Br]Br, *i.e.*, structure **I**, labeled **A** in the profile of Fig. 7. Interestingly, this species is observed experimentally if the reaction is performed in the presence of CO. In order to obtain complex [Mo(PNP^Me^-iPr)(CO)Br_2_] with structure **III**, the initial complex (**A**) has to lose two CO ligands and one bromide has to be added to the metal center. The first step of the mechanism corresponds to the loss of the first CO ligand from **A**. There are two possibilities for that process to occur. The CO ligand lost can be one of the two equatorial CO ligands (the equatorial plane being defined by the PNP ligand) or, alternatively, it can be the axial one, *trans* to the Br-ligand in **A**. Both processes were explored and are represented in Fig. 7. Loss of the axial CO leads to complex **B**, and the corresponding barrier has a high value of 30 kcal mol^–1^, corresponding to the transition state **TS_AB_**. On the other hand, dissociation of one equatorial CO ligand, from **A** to **C** presents a barrier significantly lower (19 kcal mol^–1^, **TS_AC_**) and, thus, will be the most favourable process. In the last step, from **D** to **E**,^9^ the bromine counter ion coordinates the metal producing the neutral seven-coordinate complex [Mo(PNP^Me^-iPr)(CO)_2_Br_2_] with structure **II** (**E**, in Fig. 7). This process occurs smoothly with a negligible barrier of only 1 kcal mol^–1^ (**TS_DE_**). In short, formation of **E**, from **A**, is a rather facile process, plausible to happen under the experimental conditions, with an overall barrier of 19 kcal mol^–1^ and a favourable free energy balance of Δ*G* = –10 kcal mol^–1^.

In order to obtain the observed product, [Mo(PNP^Me^-iPr)(CO)Br_2_] with structure **III**, there must be a second CO loss from the metal center and this must happen before bromide coordination, because once the Br^–^ coordinates with the metal, [Mo(PNP^Me^-iPr)(CO)_2_Br_2_] (**E**, with structure **II**) is formed which is the thermodynamic product, considerably more stable than the six-coordinate species. Thus, in the reaction path leading to [Mo(PNP^Me^-iPr)(CO)Br_2_] there will be a second CO loss following the one that yielded intermediate **C** in the previous profile.

The profile for the formation of complex [Mo(PNP^Me^-iPr)(CO)Br_2_] is represented in Fig. 8. The path starts with **F**, corresponding to **C** without the free CO molecule.^9^ From **F**, loss of another CO ligand leads to **G**, an intermediate with a five-coordinated cationic complex in the ion pair [Mo(PNP^Me^-iPr)(CO)Br]Br. This process has a high barrier energy of Δ*G* ^≠^ = 35 kcal mol^–1^ (**TS_FG_**, relative to **A**) and is clearly unfavourable from the thermodynamic point of view (Δ*G* = 22 kcal mol^–1^). **G** rearranges to **H** and, in a final step, adds Br^–^ to yield the product, [Mo(PNP^Me^-iPr)(CO)Br_2_] (**J**).^9^ This is a facile process with a barrier energy of only 1 kcal mol^–1^ (**TS_HI_**) and a free energy balance of Δ*G* = –20 kcal mol^–1^. Overall, the reaction path leading to the 6-coordinate complex **J** presents a barrier energy of 35 kcal mol^–1^ and is endergonic with Δ*G* = 14 kcal mol^–1^.

## Conclusion

We carried out a comparative study of a series of halocarbonyl Mo(ii) and W(ii) complexes of the types [M(PNP)(CO)_3_X]X and [M(PNP)(CO)_2_X_2_] (M = W, Mo; X = I, Br), featuring PNP pincer ligands based on a 2,6-diaminopyridine scaffold. The synthesis of these complexes was accomplished by treatment of [Mo(PNP)(CO)_3_] with stoichiometric amounts of I_2_ and Br_2_, respectively. The modification of the PNP ligand by introducing NMe and NPh instead of NH spacers between the aromatic pyridine ring and the P-atoms with concomitant modification of the phosphine moieties changed the steric and electronic properties of the PNP ligand significantly and led to different types of halocarbonyl complexes. While in the case of NH linkers exclusively cationic seven-coordinate complexes of the type [M(PNP)(CO)_3_X]^+^ were obtained, with NMe and NPh spacers neutral seven-coordinate complexes of the type [M(PNP)(CO)_2_X_2_] were afforded. In the case of the latter, when the reaction is performed in the presence of CO also [M(PNP)(CO)_3_X]^+^ complexes are formed which slowly lose CO to give [M(PNP)(CO)_2_X_2_]. Upon treatment of [Mo(PNP)(CO)I_2_] and [W(PNP)(CO)_2_I_2_] with a Ag^+^ salt in the presence of CO, [Mo(PNP^Me^-iPr)(CO)_3_I]^+^ and [W(PNP^Me^-iPr)(CO)_3_I]^+^, respectively, are quantitatively formed again. These experiments clearly suggest that complexes of the type [M(PNP)(CO)_3_X]^+^ are intermediates on the way to complexes of the types [M(PNP)(CO)X_2_] and [M(PNP)(CO)_2_X_2_]. By and large, the halocarbonyl tungsten chemistry parallels that of molybdenum. The only exception is molybdenum in conjunction with the PNP^Me^-iPr ligand, where the coordinatively unsaturated complex [Mo(PNP^Me^-iPr)(CO)X_2_] is formed. DFT mechanistic studies reveal that the seven-coordinate complexes should be thermodynamic as well as kinetic products. The fact that [Mo(PNP^Me^-iPr)(CO)X_2_] is the observed product suggests that the reaction follows an alternative path, for example, one involving radical intermediates. This and other possibilities will be the subject of future mechanistic studies.

## Experimental section

### General

All manipulations were performed under an inert atmosphere of argon by using Schlenk techniques. The solvents were purified according to standard procedures.^10^ The complexes [Mo(PNP)(CO)_3_] (**1a–d**), [W(PNP)(CO)_3_] (**2a–d**), and [Mo(PNP^Me^-iPr)(CO)I_2_] (**11**) were prepared according to the literature.^6,11^ The deuterated solvents were purchased from Aldrich and dried over 4 Å molecular sieves. ^1^H, ^13^C{^1^H}, and ^31^P{^1^H} NMR spectra were recorded on Bruker AVANCE-250, AVANCE-300 DPX, and AVANCE-400 spectrometers. ^1^H and ^13^C{^1^H} NMR spectra were referenced internally to residual protio-solvent, and solvent resonances, respectively, and are reported relative to tetramethylsilane (*δ* = 0 ppm). ^31^P{^1^H} NMR spectra were referenced externally to H_3_PO_4_ (85%) (*δ* = 0 ppm).

All mass spectrometric measurements were performed on an Esquire 3000^plus^ 3D-quadrupole ion trap mass spectrometer (Bruker Daltonics, Bremen, Germany) in positive-ion mode electrospray ionization (ESI-MS). Mass calibration was performed with a commercial mixture of perfluorinated trialkyl-triazines (ES Tuning Mix, Agilent Technologies, Santa Clara, CA, USA). All analytes were dissolved in CH_3_CN “LiChrosolv” quality (Merck, Darmstadt, Germany) to a concentration of roughly 1 mg mL^–1^ and doped with sodium halides (Merck, Darmstadt, Germany) to avoid or suppress dissociation of halogen substituents from the complexes. Direct infusion experiments were carried out using a Cole Parmer model 74900 syringe pump (Cole Parmer Instruments, Vernon Hills, IL, USA) at a flow rate of 2 μl min^–1^. Full scan and MS/MS-scans were measured in the range *m*/*z* 100–1000 with the target mass set to *m*/*z* 800. Further experimental conditions include: drying gas temperature: 150 °C; capillary voltage: –4 kV; skimmer voltage: 40 V; octapole and lens voltages: according to the target mass set. Helium was used as a buffer gas for full scans and as a collision gas for MS/MS-scans in the low energy CID (collision induced dissociation) mode. The activation and fragmentation width for tandem mass spectrometric (MS/MS) experiments was set to 10–12 Da to cover the entire isotope cluster for fragmentation. The corresponding fragmentation amplitude ranged from 0.3 to 0.8 V in order to maintain a low abundant precursor ion intensity in the resulting MS/MS spectrum. All mass calculations are based on the lowest mass isotope for molybdenum (^92^Mo-isotope) and tungsten (^180^W-isotope). Mass spectra and tandem spectra were averaged during data acquisition time of 1 to 2 min and one analytical scan consisted of five successive micro scans resulting in 50 and 100 analytical scans, respectively, for the final mass spectrum or MS/MS spectrum.

### Synthesis

#### General synthetic procedure for halocarbonyl molybdenum and tungsten complexes

A solution of [M(PNP)(CO)_3_] (M = Mo (**1a–d**) and W (**2a–d**)) (0.300 mmol) in CH_2_Cl_2_ (10 mL) was cooled to –78 °C and 1 equiv. of Br_2_ (0.300 mmol) was added. In the case of I_2_, the reaction was performed at room temperature. The solution was slowly warmed to room temperature and stirred for 18 h. After this period the solution was filtered, the solvent was removed under vacuum, and the yellow solid was washed twice with Et_2_O and *n*-pentane and then dried under vacuum.

#### [Mo(PNP-Cy)(CO)_3_I]I (**3a**)

The product was obtained as an orange-red solid in 87% yield. C, H, N analysis calculated for C_32_H_49_I_2_MoN_3_O_3_P_2_ (935.48): C, 41.09; H, 5.28; N, 4.49. Found: C, 40.98; H, 5.33; N, 4.57. ^1^H NMR (*δ*, CD_2_Cl_2_, 20 °C): 7.94 (s, 2H, NH), 7.03 (br, 1H, py^4^), 6.98 (br, 2H, py^3,5^), 1.94–1.32 (m, 22H, Cy), 1.28–0.86 (m, 22H, Cy). ^13^C{^1^H} NMR (*δ*, CD_2_Cl_2_, 20 °C): 232.8 (t, *J* = 19.5 Hz, CO), 215.5 (t, *J* = 13.0 Hz, CO), 160.2 (py^2,6^), 142.6 (py^4^), 103.0 (py^3,5^), 42.6 (vt, *J* = 10.8 Hz, Cy), 41.6 (vt, *J* = 11.5 Hz, Cy), 30.1–28.1 (Cy), 27.3–26.5 (Cy), 26.1–25.6 (Cy). ^31^P{^1^H} NMR (*δ*, CD_2_Cl_2_, 20 °C): 109.0. IR (ATR, cm^–1^): 2027 (*ν*_CO_), 1964 (*ν*_CO_), 1928 (*ν*_CO_). ESI-MS (*m*/*z*, CH_3_CN) positive ion: 804.0 [M]^+^, 776.0 [M – CO]^+^, 748.1 [M – 2CO]^+^.

#### [Mo(PNP-Cy)(CO)_3_Br]Br (**3b**)

The product was obtained as a yellow solid in 85% yield. C, H, N analysis calculated for C_32_H_49_Br_2_MoN_3_O_3_P_2_ (841.48): C, 45.68; H, 5.87; N, 4.99. Found: C, 45.57; H, 5.93; N, 5.20. ^1^H NMR (*δ*, CD_2_Cl_2_, 20 °C): 9.03 (s, 2H, NH), 7.20 (br, 3H, py^4^, py^3,5^), 2.03–1.60 (m, 22H, Cy), 1.48–1.24 (m, 22H, Cy). ^13^C{^1^H} NMR (*δ*, CD_2_Cl_2_, 20 °C): 234.8 (t, *J* = 17.0 Hz, CO), 218.2 (t, *J* = 13.9 Hz, CO), 160.2 (py^2,6^), 142.5 (vt, *J* = 16.6 Hz, py^4^), 102.2 (py^3,5^), 41.3 (vt, *J* = 11.7 Hz, Cy), 39.9 (vt, *J* = 10.7 Hz, Cy), 30.1–28.4 (Cy), 28.0–26.6 (Cy), 26.5–25.3 (Cy). ^31^P{^1^H} NMR (*δ*, CD_2_Cl_2_, 20 °C): 109.6. IR (ATR, cm^–1^): 2037 (*ν*_CO_), 1970 (*ν*_CO_), 1936 (*ν*_CO_).

#### [W(PNP-Cy)(CO)_3_I]I (**4a**)

The product was obtained as an orange-red solid in 89% yield. C, H, N analysis calculated for C_32_H_49_I_2_N_3_O_3_P_2_W (1023.36): C, 37.56; H, 4.83; N, 4.11. Found: C, 37.64; H, 4.88; N, 4.00. ^1^H NMR (*δ*, CD_2_Cl_2_, 20 °C): 8.25 (s, 2H, NH), 7.31 (t, *J* = 7.8 Hz, 1H, py^4^), 7.20 (d, *J* = 7.6 Hz, 2H, py^3,5^), 2.00–1.54 (m, 22H, Cy), 1.49–1.20 (m, 22H, Cy). ^13^C{^1^H} NMR (*δ*, CD_2_Cl_2_, 20 °C): 224.9 (t, *J* = 11.1 Hz, CO), 208.7 (t, *J* = 10.7 Hz, CO), 160.9 (vt, *J* = 4.8 Hz, py^2,6^), 142.8 (py^4^), 102.2 (py^3,5^), 41.7 (vt, *J* = 11.1 Hz, Cy), 41.4 (vt, *J* = 13.0 Hz, Cy), 30.2–27.9 (Cy), 27.0–26.3 (Cy), 25.8–25.5 (Cy). ^31^P{^1^H} NMR (*δ*, CD_2_Cl_2_, 20 °C): 88.4 (^1^ *J*_w–p_ = 173 Hz). IR (ATR, cm^–1^): 2022 (*ν*_CO_), 1951 (*ν*_CO_), 1915(*ν*_CO_). ESI-MS (*m*/*z*, CH_3_CN) positive ion: 894.0 [M]^+^, 866.0 [M – CO]^+^, 838.1 [M – 2CO]^+^.

#### [W(PNP-Cy)(CO)_3_Br]Br (**4b**)

The product was obtained as a yellow-orange solid in 85% yield. C, H, N analysis calculated for C_32_H_49_Br_2_N_3_O_3_P_2_W (929.36): C, 41.36; H, 5.31; N, 4.52. Found: C, 41.47; H, 5.23; N, 4.60. ^1^H NMR (*δ*, CD_2_Cl_2_, 20 °C): 9.33 (s, 2H, NH), 7.18 (br, 3H, py^4^, py^3,5^), 1.97–1.54 (m, 22H, Cy), 1.47–1.18 (m, 22H, Cy). ^13^C{^1^H} NMR (*δ*, CD_2_Cl_2_, 20 °C): 224.3 (t, *J* = 10.3 Hz, CO), 212.1 (t, *J* = 9.8 Hz, CO), 161.1 (vt, *J* = 4.9 Hz, py^2,6^), 142.3 (py^4^), 101.7 (py^3,5^), 41.1 (vt, *J* = 11.0 Hz, Cy), 39.0 (vt, *J* = 9.8 Hz, Cy), 29.5–28.7 (Cy), 27.6–26.3 (Cy), 26.0–25.5 (Cy). ^31^P{^1^H} NMR (*δ*, CD_2_Cl_2_, 20 °C): 90.7 (^1^ *J*_w–p_ = 185 Hz). IR (ATR, cm^–1^): 2024 (*ν*_CO_), 1948 (*ν*_CO_), 1911 (*ν*_CO_).

#### [W(PNP^Me^-Ph)(CO)_2_I_2_] (**5a**)

The product was obtained as a yellow solid in 89% yield. C, H, N analysis calculated for C_33_H_29_I_2_N_3_O_2_P_2_W (999.21): C, 39.67; H, 2.93; N, 4.21. Found: C, 39.56; H, 3.03; N, 4.18. ^1^H NMR (*δ*, CD_2_Cl_2_, 20 °C): 7.81 (t, *J* = 8.2 Hz, 1H, py^4^), 7.59–7.51 (m, 4H, Ph), 7.43 (t, *J* = 6.9 Hz, 2H, Ph), 7.33 (t, *J* = 7.0 Hz, 4H, Ph), 6.98 (t, *J* = 7.3 Hz, 2H, Ph), 6.78 (t, *J* = 6.9 Hz, 4H, Ph), 6.51 (t, *J* = 8.7 Hz, 4H, Ph), 6.28 (d, *J* = 8.3 Hz, 2H, py^3,5^), 3.12 (d, *J* = 5.0, 6H, NCH_3_). ^13^C{^1^H} NMR (*δ*, CD_2_Cl_2_, 20 °C): 246.9 (t, *J* = 29.2 Hz, CO), 222.2 (t, *J* = 7.0 Hz, CO), 163.0 (vt, *J* = 8.3 Hz, py^2,6^), 144.0 (Ph), 138.4 (vt, *J* = 5.9 Hz, py^4^), 132.1 (Ph), 130.1 (vdd, *J* = 9.4 Hz, *J* = 4.4 Hz, Ph), 128.3 (vt, *J* = 5.0 Hz, Ph), 127.8 (vt, *J* = 5.6 Hz, Ph), 99.7 (py^3,5^), 36.1 (vt, *J* = 3.0 Hz, NCH_3_). ^31^P{^1^H} NMR (*δ*, CD_2_Cl_2_, 20 °C): 108.0 (^1^ *J*_w–p_ = 267 Hz). IR (ATR, cm^–1^): 1952 (*ν*_CO_), 1850 (*ν*_CO_). ESI-MS (*m*/*z*, CH_3_CN, NaI) positive ion: 1019.8 [M + Na]^+^, 991.9 [M + Na – CO]^+^, 869.9 [M – I]^+^, 841.9 [M – (I + CO)]^+^.

#### [W(PNP^Me^-Ph)(CO)_2_Br_2_] (**5b**)

The product was obtained as a yellow solid in 88% yield. C, H, N analysis calculated for C_33_H_29_Br_2_N_3_O_2_P_2_W (905.21): C, 43.79; H, 3.23; N, 4.64. Found: C, 43.88; H, 3.13; N, 4.70. ^1^H NMR (*δ*, CD_2_Cl_2_, 20 °C): 7.85 (t, *J* = 8.2 Hz, 1H, py^4^), 7.70–7.55 (m, 4H, Ph), 7.52–7.37 (m, 6H, Ph), 7.15 (t, *J* = 7.4 Hz, 2H, Ph), 6.94 (t, *J* = 6.9 Hz, 4H, Ph), 6.61 (t, *J* = 8.6 Hz, 4H, Ph), 6.38 (d, *J* = 8.3 Hz, 2H, py^3,5^), 3.12 (d, *J* = 5.0 Hz, 6H, NCH_3_). ^13^C{^1^H} NMR (*δ*, CD_2_Cl_2_, 20 °C): 249.0 (t, *J* = 12.7 Hz, CO), 223.8 (t, *J* = 9.1 Hz, CO), 162.2 (vt, *J* = 8.3 Hz, py^2,6^), 143.8 (Ph), 137.1 (vt, *J* = 5.8 Hz, py^4^), 131.9 (Ph), 130.6–130.2 (Ph), 128.3 (vt, *J* = 4.9 Hz, Ph), 127.9 (vt, *J* = 5.6 Hz, Ph), 99.9 (vt, *J* = 2.4 Hz, py^3,5^), 36.1 (vt, *J* = 2.7 Hz, NCH_3_). ^31^P{^1^H} NMR (*δ*, CD_2_Cl_2_, 20 °C): 112.7 (^1^ *J*_w–p_ = 260 Hz). IR (ATR, cm^–1^): 1953 (*ν*_CO_), 1859 (*ν*_CO_).

#### [Mo(PNP^Ph^-Et)(CO)_2_I_2_] (**6a**)

The product was obtained as a yellow solid in 96% yield. C, H, N analysis calculated for C_27_H_33_I_2_MoN_3_O_2_P_2_ (843.30): C, 38.46; H, 3.94; N, 4.98. Found: C, 38.37; H, 4.10; N, 4.88. ^1^H NMR (*δ*, CD_2_Cl_2_, 20 °C): 8.2 (d, *J* = 6.9 Hz, 2H, Ph), 7.53–7.46 (m, 2H, Ph), 7.45–7.34 (m, 4H, Ph), 7.08 (vt, *J* = 8.1 Hz, 3H, Ph, py^4^), 5.39 (d, *J* = 8.1 Hz, 2H, py^3,5^), 3.22–3.01 (m, 2H, CH_2_), 2.49–2.25 (m, 4H, CH_2_), 1.98–1.85 (m, 2H, CH_2_), 1.17 (dt, *J* = 14.2 Hz, *J* = 7.4, 6H, CH_3_), 1.10–0.94 (m, 6H, CH_3_). ^13^C{^1^H} NMR (*δ*, CD_2_Cl_2_, 20 °C): 258.3 (t, *J* = 4.2 Hz, CO), 224.3 (t, *J* = 4.8 Hz, CO), 162.6 (vt, *J* = 8.2 Hz, py^2,6^), 142.4 (Ph), 138.1 (py^4^), 131.1 (Ph), 130.7 (Ph), 129.9 (Ph), 129.6 (Ph), 129.1 (Ph), 101.0 (py^3,5^), 26.0 (vt, *J* = 9.2 Hz, CH_2_), 24.0–23.4 (CH_2_), 9.1 (CH_3_), 7.1 (CH_3_). ^31^P{^1^H} NMR (*δ*, CD_2_Cl_2_, 20 °C): 133.3. IR (ATR, cm^–1^): 1974 (*ν*_CO_), 1850 (*ν*_CO_). ESI-MS (*m*/*z*, CH_3_CN, NaI) positive ion: 861.8 [M + Na]^+^, 833.8 [M + Na – CO]^+^, 711.8 [M – I]^+^, 683.9 [M – (I + CO)]^+^.

#### [Mo(PNP^Ph^-Et)(CO)_2_Br_2_] (**6b**)

The product was obtained as a yellow solid in 94% yield. C, H, N analysis calculated for C_27_H_33_Br_2_MoN_3_O_2_P_2_ (749.30): C, 43.28; H, 4.44; N, 5.61. Found: C, 43.27; H, 4.53; N, 5.80. ^1^H NMR (*δ*, CD_2_Cl_2_, 20 °C): 8.0 (d, *J* = 8.0 Hz, 2H, Ph), 7.51–7.47 (m, 2H, Ph), 7.44–7.36 (m, 4H, Ph), 7.09–7.04 (m, 3H, Ph, py^4^), 5.39 (d, *J* = 8.2 Hz, 2H, py^3,5^), 2.94–2.82 (m, 2H, CH_2_), 2.31–2.23 (m, 4H, CH_2_), 1.67–1.54 (m, 2H, CH_2_), 1.20–1.12 (m, 6H, CH_3_), 1.02–0.93 (m, 6H, CH_3_). ^13^C{^1^H} NMR (*δ*, CD_2_Cl_2_, 20 °C): 263.0 (t, *J* = 41.3 Hz, CO), 225.0 (t, *J* = 8.9 Hz, CO), 161.8 (vt, *J* = 8.2 Hz, py^2,6^), 142.1 (Ph), 138.0 (vt, *J* = 3.3 Hz, py^4^), 131.1 (Ph), 130.7 (Ph), 129.7 (Ph), 129.1 (Ph), 128.9 (Ph), 100.8 (py^3,5^), 25.6 (vt, *J* = 10.4 Hz, CH_2_), 21.3 (vt, *J* = 18.5 Hz, CH_2_), 8.4 (vt, *J* = 2.4 Hz, CH_3_), 7.2 (vt, *J* = 3.5 Hz, CH_3_). ^31^P{^1^H} NMR (*δ*, CD_2_Cl_2_, 20 °C): 138.1. IR (ATR, cm^–1^): 1975 (*ν*_CO_), 1848 (*ν*_CO_).

#### [W(PNP^Ph^-Et)(CO)_2_I_2_] (**7a**)

The product was obtained as a yellow solid in 90% yield. C, H, N analysis calculated for C_27_H_33_I_2_N_3_O_2_P_2_W (931.18): C, 34.83; H, 3.57; N, 4.51. Found: C, 34.90; H, 3.66; N, 4.41. ^1^H NMR (*δ*, CD_2_Cl_2_, 20 °C): 8.3 (d, *J* = 7.0 Hz, 2H, Ph), 7.58–7.49 (m, 4H, Ph), 7.45–7.34 (m, 1H, py^4^), 7.28–7.17 (m, 4H, Ph), 5.50 (d, *J* = 8.1 Hz, 2H, py^3,5^), 3.38–3.11 (m, 2H, CH_2_), 2.66–2.40 (m, 4H, CH_2_), 2.19–1.94 (m, 2H, CH_2_), 1.37–1.24 (m, 6H, CH_3_), 1.16–1.00 (m, 6H, CH_3_). ^13^C{^1^H} NMR (*δ*, CD_2_Cl_2_, 20 °C): 227.6 (t, *J* = 26.1 Hz, CO), 205.9 (t, *J* = 13.7 Hz, CO), 163.3 (vt, *J* = 7.3 Hz, py^2,6^), 142.1 (Ph), 138.1 (py^4^), 130.8 (Ph), 130.6 (Ph), 129.8 (Ph), 129.5 (Ph), 129.0 (Ph), 100.7 (py^3,5^), 26.1–25.4 (CH_2_), 24.0–22.8 (CH_2_), 9.1 (CH_3_), 7.3 (CH_3_). ^31^P{^1^H} NMR (*δ*, CD_2_Cl_2_, 20 °C): 109.5 (^1^ *J*_w–p_ = 250 Hz). IR (ATR, cm^–1^): 1958 (*ν*_CO_), 1826 (*ν*_CO_). ESI-MS (*m*/*z*, CH_3_CN, NaI) negative ion: 1055.8 [M + I]^–^.

#### [W(PNP^Ph^-Et)(CO)_2_Br_2_] (**7b**)

The product was obtained as a yellow solid in 88% yield. C, H, N analysis calculated for C_27_H_33_Br_2_N_3_O_2_P_2_W (837.18): C, 38.74; H, 3.97; N, 5.02. Found: C, 39.00; H, 4.03; N, 5.11. ^1^H NMR (*δ*, CD_2_Cl_2_, 20 °C): 8.6 (d, *J* = 7.0 Hz, 2H, Ph), 7.65–7.47 (m, 4H, Ph), 7.33 (t, *J* = 3.0 Hz, 1H, py^4^), 7.24–7.16 (m, 4H, Ph), 5.51 (d, *J* = 8.2 Hz, 2H, py^3,5^), 3.12–2.86 (m, 2H, CH_2_), 2.58–2.27 (m, 4H, CH_2_), 2.91–1.62 (m, 2H, CH_2_), 1.38–1.18 (m, 6H, CH_3_), 1.16–0.99 (m, 6H, CH_3_). ^13^C{^1^H} NMR (*δ*, CD_2_Cl_2_, 20 °C): 256.4 (t, *J* = 32.7 Hz, CO), 222.6 (t, *J* = 18.3 Hz, CO), 162.6 (vt, *J* = 7.7 Hz, py^2,6^), 141.9 (Ph), 138.1 (py^4^), 130.9 (Ph), 130.7 (Ph), 129.8 (Ph), 129.1 (Ph), 128.9 (Ph), 100.7 (py^3,5^), 26.3–24.7 (CH_2_), 22.4–20.7 (CH_2_), 8.5 (CH_3_), 7.7 (CH_3_). ^31^P{^1^H} NMR (*δ*, CD_2_Cl_2_, 20 °C): 115.4 (^1^ *J*_w–p_ = 244 Hz). IR (ATR, cm^–1^): 1952 (*ν*_CO_), 1823 (*ν*_CO_).

#### [W(PNP^Me^-iPr)(CO)_2_I_2_] (**8a**)

The product was obtained as a yellow solid in 83% yield. C, H, N analysis calculated for C_21_H_37_I_2_N_3_O_2_P_2_W (863.14): C, 29.22; H, 4.32; N, 4.87. Found: C, 29.17; H, 4.40; N, 4.90. ^1^H NMR (*δ*, CD_2_Cl_2_, 20 °C): 7.64 (t, *J* = 8.3 Hz, 1H, py^4^), 6.06 (d, *J* = 8.3 Hz, 2H, py^3,5^), 3.98–3.71 (m, 2H, CH), 3.21 (d, *J* = 3.9 Hz, 6H, NCH_3_), 2.99–2.70 (m, 2H, CH), 1.65 (dd, *J* = 11.4 Hz, *J* = 7.2 Hz, 6H, CH_3_), 1.58–1.29 (m, 12H, CH_3_), 1.21 (dd, *J* = 12.8 Hz, *J* = 7.4 Hz, 6H, CH_3_). ^13^C{^1^H} NMR (*δ*, CD_2_Cl_2_, 20 °C): 225.3 (t, *J* = 13.5 Hz, CO), 204.2 (br, CO), 162.4 (vt, *J* = 7.2 Hz, py^2,6^), 143.1 (py^4^), 99.0 (py^3,5^), 38.1 (vt, *J* = 9.9 Hz, CH), 36.6 (t, *J* = 3.0 Hz, NCH_3_), 31.0 (vt, *J* = 14.4 Hz, CH), 22.5 (vt, *J* = 5.4 Hz, CH_3_), 20.0 (CH_3_), 19.8 (vt, *J* = 3.1 Hz, CH_3_), 17.6 (CH_3_). ^31^P{^1^H} NMR (*δ*, CD_2_Cl_2_, 20 °C): 123.9 (^1^ *J*_w–p_ = 253 Hz). IR (ATR, cm^–1^): 1946 (*ν*_CO_), 1823 (*ν*_CO_). ESI-MS (*m*/*z*, CH_3_CN, NaI) negative ion: 987.9 [M + I]^–^.

#### [W(PNP^Me^-iPr)(CO)_2_Br_2_] (**8b**)

The product was obtained as a yellow solid in 81% yield. C, H, N analysis calculated for C_21_H_37_Br_2_N_3_O_2_P_2_W (769.14): C, 32.79; H, 4.85; N, 5.46. Found: C, 32.80; H, 4.91; N, 5.53. ^1^H NMR (*δ*, CD_2_Cl_2_, 20 °C): 7.57 (t, *J* = 8.2 Hz, 1H, py^4^), 6.04 (d, *J* = 8.2 Hz, 2H, py^3,5^), 3.48–3.33 (m, 2H, CH), 3.13 (d, *J* = 3.9 Hz, 6H, NCH_3_), 2.80–2.56 (m, 2H, CH), 1.53 (dd, *J* = 12.2 Hz, *J* = 7.2 Hz, 6H, CH_3_), 1.39 (dd, *J* = 20.3 Hz, *J* = 7.5 Hz, 6H, CH_3_), 1.24–1.08 (m, 12H, CH_3_). ^13^C{^1^H} NMR (*δ*, CD_2_Cl_2_, 20 °C): 228.3 (t, *J* = 11.9 Hz, CO), 210.1 (t, *J* = 11.0 Hz, CO), 161.8 (vt, *J* = 6.8 Hz, py^2,6^), 143.0 (py^4^), 98.9 (py^3,5^), 37.3 (vt, *J* = 9.3 Hz, CH), 36.4 (t, *J* = 2.9 Hz, NCH_3_), 28.5 (vt, *J* = 14.6 Hz, CH), 22.8 (vt, *J* = 4.5 Hz, CH_3_), 19.7 (vt, *J* = 2.2 Hz, CH_3_), 19.4 (CH_3_), 17.7 (CH_3_). ^31^P{^1^H} NMR (*δ*, CD_2_Cl_2_, 20 °C): 127.7 (^1^ *J*_w–p_ = 247 Hz). IR (ATR, cm^–1^): 1952 (*ν*_CO_), 1815 (*ν*_CO_).

#### [Mo(PNP^Me^-iPr)(CO)_3_I]I (**9**)

A solution of [Mo(PNP^Me^-iPr)(CO)_3_] (0.18 mmol, 100 mg) in CH_2_Cl_2_ (10 mL) was treated with 1 equiv. of I_2_ (0.18 mmol, 46 mg) in a CO atmosphere. The solution was stirred for 18 h and after this period the solvent was removed under vacuum, and the red-brown solid was washed twice with Et_2_O and *n*-pentane and then dried under vacuum. The product was obtained as a red-brown solid in 98% yield. C, H, N analysis calculated for C_22_H_37_I_2_MoN_3_O_3_P_2_ (803.27): C, 32.90; H, 4.64; N, 5.23. Found: C, 32.80; H, 4.73; N, 5.29. ^1^H NMR (*δ*, CD_2_Cl_2_, 20 °C): 7.96 (t, *J* = 8.3 Hz, 1H, py^4^), 6.63 (d, *J* = 8.4 Hz, 2H, py^3,5^), 3.56–3.36 (m, 2H, CH), 3.32 (d, *J* = 2.7 Hz, 6H, NCH_3_), 3.24–3.02 (m, 2H, CH), 1.67–157 (m, 6H, CH_3_), 1.56–1.42 (m, 12H, CH_3_), 1.28–1.13 (m, 6H, CH_3_). ^13^C{^1^H} NMR (*δ*, CD_2_Cl_2_, 20 °C): 231.9 (br, CO), 213.8 (t, *J* = 13.8 Hz, CO), 161.3 (vt, *J* = 6.1 Hz, py^2,6^), 144.3 (py^4^), 102.9 (py^3,5^), 36.9 (br, CH), 32.7–31.7 (CH), 30.6 (t, *J* = 10.8 Hz, NCH_3_), 21.9 (CH_3_), 19.9 (CH_3_), 19.0 (CH_3_), 18.9 (CH_3_). ^31^P{^1^H} NMR (*δ*, CD_2_Cl_2_, 20 °C): 137.1. IR (ATR, cm^–1^): 2027 (*ν*_CO_), 1971 (*ν*_CO_), 1938 (*ν*_CO_).

#### [W(PNP^Me^-iPr)(CO)_3_I]I (**10**)

A solution of [W(PNP^Me^-iPr)(CO)_3_] (0.16 mmol, 100 mg) in CH_2_Cl_2_ (10 mL) was treated with 1 equiv. of I_2_ (0.16 mmol, 40 mg) in a CO atmosphere. The solution was stirred for 18 h and after this period the solvent was removed under vacuum, and the red-brown solid was washed twice with Et_2_O and *n*-pentane and then dried under vacuum. The product was obtained as a red-brown solid in 96% yield. C, H, N analysis calculated for C_22_H_37_I_2_N_3_O_3_P_2_W (891.15): C, 29.65; H, 4.19; N, 4.72. Found: C, 29.57; H, 4.20; N, 4.83. ^1^H NMR (*δ*, CD_2_Cl_2_, 20 °C): 7.95 (dt, *J* = 8.4 Hz, *J* = 1.2 Hz, 1H, py^4^), 6.66 (d, *J* = 8.4 Hz, 2H, py^3,5^), 3.53–3.35 (m, 2H, CH), 3.29 (t, *J* = 2.7 Hz, 6H, NCH_3_), 3.25–3.05 (m, 2H, CH), 1.64–1.36 (m, 18H, CH_3_), 1.24–1.06 (m, 6H, CH_3_). ^13^C{^1^H} NMR (*δ*, CD_2_Cl_2_, 20 °C): 255.2 (t, *J* = 25.9 Hz, CO), 207.2 (t, *J* = 11.0 Hz, CO), 162.8 (py^2,6^), 146.1 (py^4^), 103.2 (py^3,5^), 37.5 (br, NCH_3_), 31.3 (CH), 31.1 (CH), 19.5 (CH_3_), 19.2 (CH_3_), 17.6 (CH_3_), 17.4 (CH_3_). ^31^P{1H} NMR (*δ*, CD_2_Cl_2_, 20 °C): 117.4 (br). IR (ATR, cm^–1^): 2020 (*ν*_CO_), 1955 (*ν*_CO_), 1915 (*ν*_CO_).

#### Reaction of [Mo(PNP^Me^-iPr)(CO)I_2_] (**11**) with AgSbF_6_ in CD_2_Cl_2_. Formation of [Mo(PNP^Me^-iPr)(CO)_3_I]SbF_6_ (**9′**)

A solution of [Mo(PNP^Me^-iPr)(CO)I_2_] (0.067 mmol, 50 mg) in CD_2_Cl_2_ (10 mL) was reacted with AgSbF_6_ (0.067 mmol, 23 mg) in a CO atmosphere. The mixture was controlled for 24h by ^31^P{^1^H} NMR. NMR spectra of **9′** are identical with those of [Mo(PNP^Me^-iPr)(CO)_3_I]I (**9**).

#### Reaction of [W(PNP^Me^-iPr)(CO)I_2_] (**8a**) with AgSbF_6_ in CD_2_Cl_2_. Formation of [W(PNP^Me^-iPr)(CO)_3_I]SbF_6_ (**10′**)

A solution of [W(PNP^Me^-iPr)(CO)I_2_] (0.058 mmol, 50 mg) in CD_2_Cl_2_ (10 mL) was reacted with AgSbF_6_ (0.058 mmol, 20 mg,) in a CO atmosphere. The mixture was controlled for 24 h by ^31^P{^1^H} NMR. NMR spectra of **10′** are identical to those of [W(PNP^Me^-iPr)(CO)_3_I]I (**10**).

### Crystal structure determination

X-ray diffraction data of **5a**, **5b**·1.5CH_2_Cl_2_, **6a**·CDCl_3_, **7a**, **8a** and **10** (CCDC entries ; 1478552–1478557) were collected at *T* = 100 K in a dry stream of nitrogen on a Bruker Kappa APEX II diffractometer system using graphite-monochromatized Mo-Kα radiation (*λ* = 0.71073 Å) and fine sliced *φ*- and *ω*-scans. Data were reduced to intensity values with SAINT and an absorption correction was applied with the multi-scan approach implemented in SADABS.^12^ The crystal of **6a**·CDCl_3_ was an association of two domains with a random orientation relationship. Intensities of both domains were integrated with overlap information and single-domain intensities were reconstructed using the HKLF4 option of TWINABS. The structures were solved by charge flipping using SUPERFLIP^13^ and refined against *F* with JANA2006.^14^ Non-hydrogen atoms were refined anisotropically. The H atoms were placed in calculated positions and thereafter refined as riding on the parent atoms. Contributions of a disordered CH_2_Cl_2_ molecule to the structure factor of **7a** was removed using the SQUEEZE routine implemented in PLATON.^15^ Molecular graphics were generated with the program MERCURY.^16^

### Computational details

All calculations were performed using the Gaussian 09 software package,^17^ without symmetry constraints. The optimized geometry and the relative stability of all the complexes with different PNP ligands for the two metal atoms (M = Mo and W), presented in Scheme 7, were obtained with the B3LYP functional.^18^ This functional includes a mixture of Hartree–Fock^19^ exchange with DFT^20^ exchange–correlation, given by Becke's three parameter functional with the Lee, Yang and Parr correlation functional, which includes both local and non-local terms. The basis set used (basis b1) consisted of the Stuttgart/Dresden ECP (SDD) basis set^21^ to describe the electrons of the metal atoms, and a standard 6-31g(d,p) basis set^22^ for all other atoms. The electronic energies (*E*_b1_) obtained at the B3LYP/b1 level of theory were converted to free energy at 298.15 K and 1 atm (*G*_b1_) by using zero point energy and thermal energy corrections based on structural and vibration frequency data calculated at the same level. *G*_b1_ represents the free energy values shown in Scheme 7.

The mechanism for the formation of the Mo complexes with the PNP^Me^-iPr ligand, represented in the profiles of Fig. 7 and 8, was obtained with an improved methodology. The geometry of all the species were optimized with the PBE0 functional and a basis set (basis b1′) equivalent to b1 but with an f-polarization function added to describe the electrons of the Mo atom.^23^ The PBE0 functional uses a hybrid generalized gradient approximation (GGA), including 25% mixture of Hartree–Fock exchange with DFT exchange–correlation, given by Perdew, Burke and Ernzerhof functional (PBE),^24^ and proved to describe well weak interactions,^25^ such as the ones that may exist in a transition state. Transition state optimizations were performed with the Synchronous Transit-Guided Quasi-Newton (STQN) Method developed by Schlegel *et al.*,^26^ following extensive searches of the potential energy surface. Frequency calculations were performed to confirm the nature of the stationary points, yielding one imaginary frequency for the transition states and none for the minima. Each transition state was further confirmed by following its vibrational mode downhill on both sides and obtaining the minima presented on the energy profiles. The electronic energies (*E*_b1′_) obtained at the PBE0/b1′ level of theory were converted to free energy at 298.15 K and 1 atm (*G*_b1′_) by using zero point energy and thermal energy corrections based on structural and vibration frequency data calculated at the same level.

Single point energy calculations were performed using the M06 functional and an improved basis set (basis b2), with the geometries optimized at the PBE0/b1′ level. Basis b2 consisted of a 3-21G basis set^27^ with an added f-polarization function for Mo^23^ and a standard 6-311++G(d,p) basis set^28^ for the rest of the elements. The M06 functional is a hybrid meta-GGA functional developed by Truhlar and Zhao,^29^ and it was shown to perform very well for the kinetics of transition metal molecules, providing a good description of weak and long range interactions.^30^ Solvent effects (CH_2_Cl_2_) were considered in all calculations (geometry optimizations included) using the Polarizable Continuum Model (PCM) initially devised by Tomasi and coworkers^31^ with radii and non-electrostatic terms of the SMD solvation model, developed by Truhlar *et al.* ^32^ The free energy values presented in the profiles (*G*solnb2) were derived from the electronic energy values obtained at the M06/b2//PBE0/b1′ level (*E*solnb2) according to the following expression:*G*solnb2 = *E*solnb2 + *G*_b1′_ – *E*_b1′_.

## References

[cit1] Dilsky S. (2007). J. Organomet. Chem..

[cit2] Wingard L. A., White P. S., Templeton J. L. (2012). Dalton Trans..

[cit3] Benito-Garagorri D., Becker E., Wiedermann J., Lackner W., Pollak M., Mereiter K., Kisala J., Kirchner K. (2006). Organometallics.

[cit4] Öztopcu Ö., Holzhacker C., Puchberger M., Weil M., Mereiter K., Veiros L. F., Kirchner K. (2013). Organometallics.

[cit5] De Aguiar S. R. M. M., Stöger B., Pittenauer E., Allmaier G., Puchberger M., Veiros L. F., Kirchner K. (2014). J. Organomet. Chem..

[cit6] De Aguiar S. R. M. M., Öztopcu Ö., Stöger B., Mereiter K., Veiros L. F., Pittenauer E., Allmaier G., Kirchner K. (2014). Dalton Trans..

[cit7] (c) Morales-Morales D. and Jensen C. M., The Chemistry of Pincer Compounds, Elsevier, Amsterdam, 2007.

[cit8] Baker P. K., Al-Jahdali M., Meehan M. M. (2002). J. Organomet. Chem..

[cit9] **D** and **F** are similar to **C** without the neighbour CO molecule. The same happens with **J** relative to **I**

[cit10] Perrin D. D. and Armarego W. L. F., Purification of Laboratory Chemicals, Pergamon, New York, 3rd edn, 1988.

[cit11] Mastalir M., De Aguiar S. R. M. M., Glatz M., Stöger B., Kirchner K. (2016). Organometallics.

[cit12] Bruker computer programs: APEX2, SAINT, SADABS and TWINABS, Bruker AXS Inc., Madison, WI, 2012.

[cit13] Palatinus L., Chapuis G. (2007). J. Appl. Crystallogr..

[cit14] Petříček V., Dušek M., Palatinus L. (2014). Z. Kristallogr..

[cit15] Spek A. L. (2009). Acta Crystallogr., Sect. D: Biol. Crystallogr..

[cit16] Macrae C. F., Edgington P. R., McCabe P., Pidcock E., Shields G. P., Taylor R., Towler M., van de Streek J. (2006). J. Appl. Crystallogr..

[cit17] Frisch M. J., Trucks G. W., Schlegel H. B., Scuseria G. E., Robb M. A., Cheeseman J. R., Scalmani G., Barone V., Mennucci B., Petersson G. A., Nakatsuji H., Caricato M., Li X., Hratchian H. P., Izmaylov A. F., Bloino J., Zheng G., Sonnenberg J. L., Hada M., Ehara M., Toyota K., Fukuda R., Hasegawa J., Ishida M., Nakajima T., Honda Y., Kitao O., Nakai H., Vreven T., Montgomery Jr. J. A., Peralta J. E., Ogliaro F., Bearpark M., Heyd J. J., Brothers E., Kudin K. N., Staroverov V. N., Kobayashi R., Normand J., Raghavachari K., Rendell A., Burant J. C., Iyengar S. S., Tomasi J., Cossi M., Rega N., Millam J. M., Klene M., Knox J. E., Cross J. B., Bakken V., Adamo C., Jaramillo J., Gomperts R., Stratmann R. E., Yazyev O., Austin A. J., Cammi R., Pomelli C., Ochterski J. W., Martin R. L., Morokuma K., Zakrzewski V. G., Voth G. A., Salvador P., Dannenberg J. J., Dapprich S., Daniels A. D., Farkas Ö., Foresman J. B., Ortiz J. V., Cioslowski J. and Fox D. J., Gaussian 09, Revision A.02, Gaussian, Inc., Wallingford CT, 2009.

[cit18] Becke A. D. (1993). J. Chem. Phys..

[cit19] Hehre W. J., Radom L., Schleyer P. V. R. and Pople J. A., Ab Initio Molecular Orbital Theory, John Wiley & Sons, New York, 1986.

[cit20] Parr R. G. and Yang W., in Density Functional Theory of Atoms and Molecules, Oxford University Press, New York, 1989.

[cit21] Haeusermann U., Dolg M., Stoll H., Preuss H. (1993). Mol. Phys..

[cit22] Ditchfield R., Hehre W. J., Pople J. A. (1971). J. Chem. Phys..

[cit23] Ehlers A. W., Böhme M., Dapprich S., Gobbi A., Höllwarth A., Jonas V., Köhler K. F., Stegmann R., Veldkamp A., Frenking G. (1993). Chem. Phys. Lett..

[cit24] Perdew J. P., Burke K., Ernzerhof M. (1997). Phys. Rev. Lett..

[cit25] Zhao Y., Truhlar D. G. (2005). J. Chem. Theory Comput..

[cit26] Peng C., Yayala P., Schlegel H. B., Frisch M. J. (1996). J. Comput. Chem..

[cit27] Binkley J. S., Pople J. A., Hehre W. J. (1980). J. Am. Chem. Soc..

[cit28] McClean A. D., Chandler G. S. (1980). J. Chem. Phys..

[cit29] Zhao Y., Truhlar D. G. (2008). Theor. Chem. Acc..

[cit30] Zhao Y., Truhlar D. G. (2008). Acc. Chem. Res..

[cit31] Cancès M. T., Mennucci B., Tomasi J. (1997). J. Chem. Phys..

[cit32] Marenich A. V., Cramer C. J., Truhlar D. G. (2009). J. Phys. Chem. B.

