# Supplementary material for: Structural diversity of halocarbonyl molybdenum and tungsten PNP pincer complexes through ligand modifications
Source: Dalton Trans. 2016 Jul 20;45(35):13834–45. doi: 10.1039/c6dt02251k (PMC5048400; doi:10.1039/c6dt02251k)
Supplement: Supplementary file 1 [file DT-045-C6DT02251K-s001.pdf]

Electronic Supplementary Material (ESI) for Dalton Transactions.
This journal is © The Royal Society of Chemistry 2016

Electronic Supporting Information for

Structural Diversity of Halocarbonyl Molybdenum and Tungsten PNP Pincer Complexes through Ligand Modifications

Sara R. M. M. de Aguiar,[a] Berthold Stöger,[b] Ernst Pittenauer,[b] Günter Allmaier,[b] Luis F. Veiros,[c] and Karl Kirchner*,[a]

Atomic coordinates of all optimized species (PBE0/b1')

CO

C	0.000000	0.000000	-0.002450
O	0.000000	0.000000	1.132450

A

Mo	1.201951	0.574124	0.279129
P	-0.641168	2.265814	0.085531
P	1.975588	-1.805024	0.092327
N	-0.661167	-0.636693	-0.100782
N	-1.958980	1.234567	0.524351
N	0.665701	-2.429045	-0.836796
C	-1.892425	-0.101557	0.158440
C	-3.056494	-0.860543	0.037294
C	-2.958479	-2.161426	-0.426293
C	-1.727378	-2.700570	-0.757111
C	-0.586449	-1.916490	-0.566947
C	-0.741855	3.738399	1.214895
C	-1.045631	3.413473	2.678095
C	-1.034790	2.845279	-1.643110
C	-2.470284	3.333894	-1.829695
C	1.523035	-4.231522	1.550043
C	3.093254	-2.550003	2.602971
C	3.519212	-2.191753	-0.866321
C	4.056463	-3.618831	-0.736349
C	0.549593	0.593403	2.151699
O	0.205451	0.508414	3.249834
C	2.773666	0.681585	1.438825
O	3.662445	0.819461	2.171798
H	-4.049119	-0.483903	0.261599
H	-3.889965	-2.720500	-0.512301
H	-1.634689	-3.722482	-1.100816
H	-1.335643	4.342685	3.180237
H	-0.154442	3.036080	3.184929
H	-1.852870	2.693644	2.822312
H	-2.581154	3.686303	-2.861482
H	-2.714503	4.174089	-1.171552
H	-3.205755	2.541936	-1.678733
H	1.376489	-4.654975	2.550347
H	2.315540	-4.804521	1.062647
H	0.591869	-4.375796	0.996240
H	2.903332	-3.048852	3.559697
H	3.291247	-1.499640	2.821715
H	3.999131	-2.990164	2.177593
H	4.868230	-3.744149	-1.461458
H	3.312438	-4.391649	-0.937513
H	4.475382	-3.799887	0.257067
C	4.622060	-1.179905	-0.546392
H	4.317040	-0.155195	-0.774816
H	4.936541	-1.233386	0.500424
H	5.496009	-1.412376	-1.164477
C	-0.042406	3.874922	-2.182060
H	1.000451	3.586054	-2.037629
H	-0.203208	3.974491	-3.261015
H	-0.204151	4.860780	-1.735819
C	0.544868	4.566395	1.150362
H	0.838467	4.850068	0.138668
H	0.389121	5.488591	1.720235
H	1.374374	4.026584	1.619005
C	2.319231	2.143557	-0.200474
O	3.025640	3.004786	-0.497227
H	1.019889	-2.258420	2.178832
C	1.876975	-2.752447	1.699611
H	3.196752	-2.010537	-1.899239
H	-0.898613	1.919097	-2.216411
H	-1.563074	4.336925	0.798940
Br	1.561870	0.417732	-2.368536
Br	-6.466840	-1.872684	0.165353
C	-3.275706	1.767078	0.877242
H	-3.641579	1.272987	1.782605
H	-3.201228	2.835013	1.073367
H	-4.006522	1.620711	0.077186
C	0.766772	-3.575497	-1.731578
H	0.066591	-3.441087	-2.559955
H	1.766567	-3.619334	-2.160062
H	0.554436	-4.528798	-1.234964

TS_{AB}

Mo	1.107796	0.480038	0.314693
P	-0.743499	2.165716	0.082717
P	1.954018	-1.839068	0.146225
N	-0.681442	-0.751972	0.004686
N	-2.018054	1.111753	0.595581
N	0.663697	-2.498546	-0.797606
C	-1.928933	-0.215226	0.227806
C	-3.089818	-0.971630	0.035976
C	-2.967596	-2.270541	-0.422429
C	-1.723447	-2.812317	-0.705436
C	-0.593310	-2.022865	-0.499696
C	-0.771154	3.661114	1.191094
C	-1.035629	3.382711	2.670948
C	-1.250939	2.739629	-1.616144
C	-2.709859	3.178321	-1.735228
C	1.581385	-4.302815	1.575316
C	3.119846	-2.589376	2.624748
C	3.499863	-2.173701	-0.831862
C	4.061635	-3.595226	-0.742305
C	0.519164	0.523470	2.175266
O	0.210002	0.467571	3.294220
C	2.726460	0.640197	1.427086
O	3.608096	0.791601	2.166753
H	-4.073974	-0.515596	0.110601
H	-3.863259	-2.862892	-0.587303
H	-1.621394	-3.830258	-1.056850
H	-1.225736	4.340697	3.167515
H	-0.158496	2.939446	3.146936

H	-1.892595	2.735273	2.861966
H	-2.875854	3.546065	-2.755026
H	-2.945661	4.003586	-1.054339
H	-3.425303	2.367967	-1.565305
H	1.456900	-4.740273	2.572657
H	2.383231	-4.850295	1.073899
H	0.648080	-4.466546	1.030464
H	2.959556	-3.103685	3.578753
H	3.289530	-1.535290	2.850146
H	4.031324	-2.998343	2.180375
H	4.857689	-3.693979	-1.488778
H	3.327084	-4.378213	-0.938143
H	4.507750	-3.785804	0.237540
C	4.593439	-1.157267	-0.495428
H	4.271433	-0.130505	-0.687075
H	4.921955	-1.241184	0.544878
H	5.461318	-1.360909	-1.132227
C	-0.311655	3.815330	-2.163063
H	0.744099	3.553892	-2.048954
H	-0.506562	3.937553	-3.234233
H	-0.494366	4.783458	-1.685778
C	0.565781	4.401409	1.065958
H	0.829833	4.664513	0.040356
H	0.508431	5.331540	1.642430
H	1.375755	3.795380	1.486488
C	3.421566	3.140889	-0.312488
O	3.927407	3.811769	-1.075500
H	1.033568	-2.350518	2.233540
C	1.896136	-2.816584	1.737014
H	3.166455	-1.972695	-1.858378
H	-1.103303	1.829263	-2.212753
H	-1.571802	4.297654	0.792365
Br	1.832375	0.776241	-2.154489
Br	-6.080728	0.697678	-1.193617
C	-3.322228	1.623884	1.032070
H	-3.597234	1.145789	1.978773
H	-3.243157	2.697044	1.200772
H	-4.121190	1.451718	0.297169
C	0.781254	-3.646759	-1.688226
H	0.054906	-3.541856	-2.498069
H	1.770296	-3.653745	-2.143111
H	0.616169	-4.604405	-1.182420

B

Mo	1.118172	0.426911	0.253406
P	-0.712450	2.119360	-0.001562
P	1.922922	-1.912487	0.100974
N	-0.715321	-0.765125	-0.031472
N	-2.016706	1.113200	0.548678
N	0.603071	-2.542063	-0.825951
C	-1.949752	-0.224707	0.237914
C	-3.116516	-0.996094	0.151465
C	-3.010112	-2.309988	-0.260309
C	-1.777735	-2.859763	-0.589934
C	-0.646909	-2.056963	-0.483009
C	-0.724344	3.650794	1.055548
C	-0.988151	3.419795	2.544211
C	-1.248551	2.655453	-1.718220
C	-2.754493	2.877263	-1.856085
C	1.546183	-4.336023	1.598935
C	3.105435	-2.593789	2.579541
C	3.437730	-2.301386	-0.909217
C	3.974410	-3.730174	-0.786033
C	0.612333	0.536988	2.103498
O	0.349059	0.548395	3.237753
C	2.800972	0.643534	1.251560
O	3.727440	0.823383	1.923401
H	-4.098695	-0.545180	0.266712
H	-3.909449	-2.912703	-0.350609
H	-1.691119	-3.890714	-0.905922
H	-1.164100	4.394654	3.012372
H	-0.115382	2.979004	3.030159
H	-1.852560	2.789691	2.757246
H	-2.960409	3.177520	-2.890441
H	-3.103244	3.689867	-1.209265
H	-3.358550	1.989202	-1.651298
H	1.431296	-4.743802	2.609876
H	2.337541	-4.902313	1.102375
H	0.604511	-4.507676	1.071114
H	2.951575	-3.065112	3.556495
H	3.284009	-1.531082	2.756385
H	4.009154	-3.028013	2.143039
H	4.760553	-3.865261	-1.537079
H	3.222866	-4.503949	-0.951295
H	4.427185	-3.898268	0.194870
C	4.559991	-1.298381	-0.632853
H	4.261700	-0.271567	-0.858989
H	4.904997	-1.349095	0.404113
H	5.411473	-1.547312	-1.275673
C	-0.494866	3.881678	-2.235925
H	0.589037	3.763585	-2.188309
H	-0.768229	4.037474	-3.285648
H	-0.781853	4.785487	-1.688710
C	0.616476	4.379649	0.908779
H	0.891224	4.602885	-0.123151
H	0.560627	5.328754	1.453548
H	1.418647	3.781458	1.353843
C	4.110931	4.110021	0.863360
O	4.306963	4.926034	0.099347
H	1.015188	-2.362161	2.203351
C	1.871230	-2.848504	1.715025
H	3.083308	-2.133219	-1.935664
H	-0.964481	1.790297	-2.333243
H	-1.523874	4.275908	0.635607
Br	2.436760	1.881787	-1.384910
Br	-6.153130	0.541615	-1.052258
C	-3.301425	1.658059	1.001389
H	-3.541672	1.245012	1.987492
H	-3.215641	2.739554	1.093598
H	-4.127154	1.439000	0.310087
C	0.673539	-3.731669	-1.667854
H	-0.079749	-3.650552	-2.455486
H	1.646211	-3.773957	-2.155284
H	0.512507	-4.664443	-1.116389

TS$_{AC}$

Mo	1.133973	0.537822	0.163230
P	-0.737740	2.221134	0.034164
P	1.989522	-1.813247	0.096776
N	-0.698201	-0.724275	-0.153837
N	-2.018196	1.135348	0.467137
N	0.668472	-2.545513	-0.757697
C	-1.938236	-0.172822	0.009981
C	-3.104249	-0.890505	-0.271759
C	-2.972418	-2.191674	-0.730527
C	-1.726253	-2.770389	-0.901400
C	-0.591419	-2.007963	-0.603020
C	-0.827953	3.587139	1.283176
C	-0.709498	3.069738	2.712204
C	-1.254011	2.922809	-1.618716
C	-2.406427	3.921872	-1.517560
C	1.778986	-4.220757	1.679985
C	3.137324	-2.322142	2.643114
C	3.540840	-2.211075	-0.856693
C	4.172867	-3.585038	-0.625089
C	0.314990	0.061036	3.046504
O	0.225145	-0.195632	4.146776
C	2.636593	0.844634	1.353149
O	3.521473	1.084171	2.076114
H	-4.092492	-0.431290	-0.215299
H	-3.866433	-2.763142	-0.965819
H	-1.631161	-3.795881	-1.231040
H	-0.881242	3.899375	3.406744
H	0.298643	2.684354	2.899690
H	-1.436783	2.285552	2.941022
H	-2.745672	4.168379	-2.529872
H	-2.087952	4.855603	-1.042538
H	-3.266726	3.528930	-0.971205
H	1.627476	-4.591414	2.700527
H	2.652205	-4.733701	1.271515

H	0.900917	-4.508142	1.097191
H	2.981856	-2.764743	3.633414
H	3.236257	-1.243820	2.779822
H	4.086895	-2.705804	2.258511
H	5.001449	-3.700316	-1.332792
H	3.490330	-4.422582	-0.780704
H	4.590453	-3.669633	0.381817
C	4.583327	-1.115097	-0.614938
H	4.210228	-0.127740	-0.897328
H	4.906596	-1.085803	0.430495
H	5.466040	-1.330686	-1.227073
C	-0.098416	3.491365	-2.440285
H	0.679837	2.745459	-2.614784
H	-0.488004	3.800027	-3.417239
H	0.350085	4.373587	-1.975114
C	0.240736	4.645644	1.007387
H	0.150564	5.093577	0.015090
H	0.132682	5.449636	1.743427
H	1.248082	4.231815	1.115578
C	2.202041	2.059907	-0.404088
O	2.865181	2.951844	-0.744565
H	1.047272	-2.289126	2.179107
C	1.964983	-2.705257	1.740541
H	3.208055	-2.130459	-1.898296
H	-1.617597	2.020150	-2.129144
H	-1.811346	4.053570	1.149252
Br	1.568891	0.170011	-2.399128
Br	-6.414672	0.581236	-0.816837
C	-3.338381	1.604242	0.901072
H	-3.700905	0.951157	1.701438
H	-3.251421	2.611013	1.307746
H	-4.092068	1.598222	0.102417
C	0.801257	-3.749735	-1.568392
H	0.236283	-3.629887	-2.497449
H	1.843979	-3.895352	-1.840202
H	0.450188	-4.650437	-1.052166

C

Mo	1.135929	0.546950	0.103847
P	-0.743344	2.223256	0.012583
P	1.998182	-1.791843	0.085947
N	-0.684263	-0.726751	-0.171825
N	-2.012176	1.126740	0.452345
N	0.688613	-2.540397	-0.773685
C	-1.926909	-0.181880	-0.002607
C	-3.090042	-0.907079	-0.275972
C	-2.953828	-2.211121	-0.726286
C	-1.705686	-2.785169	-0.898168
C	-0.573806	-2.013315	-0.614038
C	-0.825419	3.574401	1.277915
C	-0.692620	3.032911	2.696591
C	-1.277218	2.937204	-1.628122
C	-2.422351	3.942103	-1.508286
C	1.675778	-4.152615	1.694434
C	3.182964	-2.338266	2.610366
C	3.555306	-2.206894	-0.847823
C	4.163716	-3.593904	-0.629779
C	0.175524	-0.009378	3.242034
O	0.265411	-0.137140	4.365418
C	2.585061	0.854280	1.353885
O	3.437339	1.091884	2.116680
H	-4.080360	-0.452655	-0.217660
H	-3.846156	-2.788792	-0.952712
H	-1.605934	-3.813561	-1.217508
H	-0.839405	3.853320	3.407693
H	0.312118	2.628696	2.860224
H	-1.427513	2.255708	2.924429
H	-2.769622	4.200580	-2.514845
H	-2.094484	4.869244	-1.026796
H	-3.279596	3.548210	-0.957574
H	1.572303	-4.524120	2.720648
H	2.481978	-4.717493	1.220200
H	0.741176	-4.371593	1.172185
H	3.008909	-2.723308	3.621306
H	3.380928	-1.268233	2.697539
H	4.086449	-2.821143	2.227995
H	4.985095	-3.719425	-1.344290
H	3.464891	-4.417568	-0.786416
H	4.587613	-3.694981	0.372665
C	4.609786	-1.127455	-0.583706
H	4.249732	-0.131077	-0.851773
H	4.927719	-1.117416	0.463696
H	5.493168	-1.343631	-1.194619
C	-0.121769	3.502266	-2.452237
H	0.650009	2.750858	-2.634613
H	-0.512507	3.821973	-3.425105
H	0.336638	4.376674	-1.981691
C	0.242151	4.635350	1.008245
H	0.144013	5.097516	0.023174
H	0.141585	5.428600	1.756968
H	1.249727	4.218357	1.101836
C	2.226537	2.063900	-0.442303
O	2.895003	2.957328	-0.768407
H	1.090172	-2.172710	2.208665
C	1.962590	-2.653233	1.745208
H	3.235146	-2.107745	-1.892011
H	-1.650392	2.039887	-2.141150
H	-1.809619	4.043278	1.160204
Br	1.606532	0.166059	-2.443783
Br	-6.405997	0.551305	-0.821914
C	-3.332489	1.587441	0.895015
H	-3.686757	0.931554	1.696796
H	-3.249008	2.594380	1.302123
H	-4.090520	1.577910	0.100464
C	0.830786	-3.745369	-1.580975
H	0.219638	-3.652963	-2.483210
H	1.863761	-3.850694	-1.904262
H	0.538913	-4.654927	-1.043649

D

Mo	1.438092	0.291968	-1.369106
P	-0.231015	1.737855	-0.238751
P	2.351405	-1.743930	-0.385677
N	-0.252387	-1.103729	-0.775132
N	-1.600548	0.688624	-0.118784
N	1.206514	-2.883774	-1.003218
C	-1.494754	-0.622376	-0.504884
C	-2.625350	-1.450371	-0.581294
C	-2.446941	-2.790867	-0.866925
C	-1.175148	-3.323002	-1.033182
C	-0.092593	-2.447047	-0.954062
C	-0.123337	2.489923	1.452763
C	0.052457	1.431577	2.536633
C	-0.781426	3.082773	-1.417832
C	-1.498627	4.288577	-0.808933
C	1.738395	-3.275786	1.938794
C	3.277024	-1.318128	2.299796
C	4.021437	-2.377219	-0.890868
C	4.539228	-3.594228	-0.121483
C	2.581365	1.047353	-0.040344
O	3.358717	1.555347	0.674964
H	-3.616428	-1.044785	-0.444215
H	-3.313645	-3.442779	-0.931402
H	-1.021903	-4.384342	-1.174047
H	0.014510	1.912893	3.520215
H	1.020690	0.927882	2.451238
H	-0.738576	0.676510	2.504278
H	-1.913430	4.880032	-1.633657
H	-0.822959	4.942548	-0.249715
H	-2.334039	4.026250	-0.155690
H	1.498826	-3.228912	3.007339
H	2.561668	-3.984926	1.818168
H	0.861169	-3.670727	1.420456
H	2.950424	-1.253250	3.343862
H	3.597673	-0.322225	1.993690
H	4.145459	-1.981374	2.274333
H	5.417684	-3.980747	-0.650280
H	3.814906	-4.407686	-0.046640
H	4.857993	-3.328692	0.889304
C	5.050965	-1.245334	-0.875092

H	4.761564	-0.422666	-1.533519
H	5.213940	-0.849376	0.131562
H	6.007306	-1.640615	-1.234263
C	0.368557	3.540579	-2.315158
H	0.715691	2.724901	-2.958161
H	0.010125	4.338780	-2.975822
H	1.222646	3.932311	-1.751722
C	0.944482	3.582218	1.548808
H	0.914189	4.292193	0.718268
H	0.783765	4.150403	2.471441
H	1.948939	3.157962	1.593313
C	2.793819	1.411076	-2.281484
O	3.616403	2.051903	-2.779865
H	1.250862	-1.215675	1.622782
C	2.117253	-1.871053	1.467726
H	3.856374	-2.651574	-1.939273
H	-1.487242	2.521520	-2.058691
H	-1.104418	2.963782	1.589474
Br	2.152108	-1.126611	-3.554220
Br	-2.672991	0.994964	-3.641357
C	-2.936262	1.234764	0.097759
H	-3.487581	0.623707	0.818458
H	-2.859022	2.234323	0.521252
H	-3.471387	1.286656	-0.859187
C	1.489907	-4.227906	-1.474233
H	0.873505	-4.439479	-2.352249
H	2.529251	-4.306109	-1.786313
H	1.292916	-4.985351	-0.705746

TS_{DE}

Mo	1.437002	0.304061	-1.358310
P	-0.249739	1.747137	-0.242436
P	2.349765	-1.739331	-0.387819
N	-0.260093	-1.095429	-0.771792
N	-1.614591	0.694426	-0.117838
N	1.201344	-2.873750	-1.008272
C	-1.502218	-0.620559	-0.491754
C	-2.629516	-1.454332	-0.545286
C	-2.449500	-2.795049	-0.828189
C	-1.177839	-3.319568	-1.013166
C	-0.098072	-2.438169	-0.948563
C	-0.145374	2.507752	1.445990
C	0.039433	1.455101	2.534156
C	-0.801534	3.090122	-1.425116
C	-1.533586	4.290427	-0.822919
C	1.734577	-3.278563	1.930919
C	3.296096	-1.340344	2.294439
C	4.016319	-2.374976	-0.902824
C	4.534606	-3.597935	-0.143517
C	2.555774	1.050435	-0.003688
O	3.323936	1.552878	0.725478
H	-3.620265	-1.054805	-0.388329
H	-3.313412	-3.452205	-0.874510
H	-1.020646	-4.380573	-1.152135
H	0.001475	1.940686	3.515657
H	1.009962	0.955956	2.449096
H	-0.747472	0.695570	2.507265
H	-1.946148	4.879181	-1.650639
H	-0.865704	4.949021	-0.259861
H	-2.371207	4.024360	-0.174206
H	1.505788	-3.237372	3.002071
H	2.549435	-3.995118	1.797074
H	0.848485	-3.660477	1.417932
H	2.974571	-1.270280	3.339733
H	3.631679	-0.349710	1.987315
H	4.153378	-2.017727	2.265596
H	5.407121	-3.985334	-0.681504
H	3.807398	-4.408767	-0.067875
H	4.862414	-3.339731	0.866145
C	5.048310	-1.245555	-0.888350
H	4.754849	-0.418961	-1.540048
H	5.219876	-0.855510	0.119183
H	6.001003	-1.640492	-1.257528
C	0.356564	3.557619	-2.307285
H	0.709238	2.745669	-2.951546
H	0.003343	4.360136	-2.965573
H	1.203408	3.947021	-1.731513
C	0.914609	3.608214	1.536715
H	0.872513	4.319912	0.708266
H	0.756155	4.172912	2.461923
H	1.922955	3.192582	1.573174
C	2.798878	1.436443	-2.243803
O	3.624811	2.086350	-2.724623
H	1.266187	-1.210546	1.630296
C	2.124440	-1.874744	1.466398
H	3.844861	-2.643757	-1.951440
H	-1.486263	2.517546	-2.076179
H	-1.129767	2.975129	1.582175
Br	2.168921	-1.088137	-3.525823
Br	-2.321815	0.784539	-3.745332
C	-2.952756	1.233149	0.093875
H	-3.493751	0.640782	0.837761
H	-2.883738	2.246907	0.482725
H	-3.503260	1.249441	-0.854545
C	1.481215	-4.217775	-1.481765
H	0.854252	-4.430293	-2.352010
H	2.516640	-4.294295	-1.807091
H	1.294877	-4.975793	-0.711154

E

Mo	-0.064342	-0.618121	-0.368716
P	-2.103038	0.787996	-0.363110
P	2.113942	0.550059	-0.330428
N	-0.031020	0.677445	1.491320
N	-2.336040	0.929040	1.356412
N	2.292593	0.756341	1.395750
C	-1.203046	0.950588	2.125033
C	-1.236501	1.271677	3.489313
C	-0.028720	1.354731	4.165411
C	1.179582	1.176417	3.508608
C	1.146263	0.862721	2.141764
C	-2.184723	2.588945	-0.873978
C	-1.452991	3.534438	0.074270
C	-3.685572	0.060923	-1.060946
C	-4.810095	1.049188	-1.371641
C	3.103414	3.247886	-0.207528
C	2.390041	2.416812	-2.480005
C	3.647407	-0.354883	-0.902490
C	4.940184	0.444274	-1.085373
C	-0.035776	0.017646	-2.178640
O	-0.035652	0.260504	-3.328144
H	-2.172065	1.470175	3.994115
H	-0.027023	1.601704	5.223745
H	2.117654	1.303806	4.030588
H	-1.569628	4.561135	-0.292083
H	-0.381466	3.328594	0.131289
H	-1.862014	3.497759	1.087495
H	-5.702539	0.476098	-1.647609
H	-4.559980	1.690180	-2.223442
H	-5.086041	1.694096	-0.532750
H	2.936746	4.271750	-0.563039
H	4.157797	3.012910	-0.375359
H	2.914132	3.238962	0.868939
H	2.169977	3.444447	-2.791917
H	1.745115	1.753518	-3.058356
H	3.426777	2.211600	-2.757156
H	5.757962	-0.269302	-1.239029
H	5.213094	1.068991	-0.233188
H	4.903224	1.084448	-1.969980
C	3.335137	-1.123946	-2.189595
H	2.513486	-1.829678	-2.050438
H	3.087728	-0.452327	-3.018197
H	4.224018	-1.694259	-2.482923
C	-3.377693	-0.772347	-2.306823
H	-2.676470	-1.579124	-2.086330
H	-4.308624	-1.222301	-2.670149
H	-2.969314	-0.160070	-3.117416
C	-1.765093	2.809751	-2.324659
H	-2.253781	2.118030	-3.017171
H	-2.032289	3.828542	-2.628131

H	-0.683663	2.700326	-2.444372
H	1.142092	2.629120	-0.768864
C	2.170327	2.307375	-0.969164
H	3.784179	-1.093709	-0.105086
H	-4.004142	-0.628889	-0.272166
H	-3.252093	2.823651	-0.787287
Br	1.570007	-2.386685	0.854850
Br	-1.946696	-2.218664	0.750309
C	-0.117575	-2.173111	-1.560301
O	-0.146258	-3.089287	-2.268675
C	3.565171	0.766499	2.098740
H	3.571810	0.000237	2.881300
H	4.373747	0.538919	1.410856
H	3.769697	1.742977	2.553538
C	-3.622314	1.140140	1.993162
H	-4.422921	0.974303	1.275789
H	-3.761477	0.424632	2.810440
H	-3.719964	2.158794	2.389536

F

Mo	1.487389	0.188886	-1.247363
P	-0.205629	1.612993	-0.129000
P	2.328763	-1.918856	-0.343817
N	-0.235705	-1.197897	-0.758547
N	-1.527300	0.541843	0.117967
N	1.174953	-3.000480	-1.052284
C	-1.468002	-0.712256	-0.443356
C	-2.629155	-1.467006	-0.654084
C	-2.480788	-2.780213	-1.072246
C	-1.223359	-3.351674	-1.219434
C	-0.111893	-2.529183	-1.029389
C	-0.030649	2.528638	1.468653
C	0.242434	1.567900	2.624157
C	-0.845737	2.804612	-1.428022
C	-1.791514	3.879792	-0.893539
C	1.619030	-3.535320	1.890799
C	3.196905	-1.636222	2.375865
C	3.999071	-2.560047	-0.839910
C	4.482680	-3.815541	-0.110849
C	2.589201	0.862291	0.161858
O	3.339677	1.322923	0.934205
H	-3.614085	-1.008803	-0.572232
H	-3.366963	-3.382051	-1.255312
H	-1.104204	-4.402895	-1.445428
H	0.241645	2.130436	3.564216
H	1.218656	1.083675	2.528519
H	-0.522787	0.789543	2.700310
H	-2.197191	4.436327	-1.746086
H	-1.275635	4.597961	-0.249220
H	-2.640061	3.466147	-0.343696
H	1.360336	-3.533014	2.955834
H	2.427664	-4.257645	1.750809
H	0.742621	-3.883244	1.338546
H	2.842019	-1.591342	3.411531
H	3.561661	-0.643805	2.111581
H	4.041052	-2.330005	2.353164
H	5.370793	-4.188285	-0.633307
H	3.748533	-4.623286	-0.090987
H	4.777103	-3.597735	0.918310
C	5.045806	-1.446694	-0.757171
H	4.778306	-0.587659	-1.377464
H	5.201625	-1.103528	0.269846
H	6.000203	-1.839749	-1.123626
C	0.276555	3.409211	-2.271990
H	0.795888	2.642392	-2.855517
H	-0.154038	4.122807	-2.983468
H	1.014711	3.945425	-1.667741
C	1.005213	3.650721	1.378675
H	0.766986	4.375531	0.596426
H	1.016557	4.189431	2.332408
H	2.013872	3.272323	1.201843
C	2.877427	1.345051	-2.059013
O	3.716338	2.009816	-2.494531
H	1.189628	-1.448787	1.658114
C	2.042212	-2.120407	1.494654
H	3.851272	-2.790003	-1.901544
H	-1.424829	2.127446	-2.070306
H	-1.007964	2.998767	1.631828
Br	2.221767	-1.150474	-3.470880
Br	-5.689570	0.472618	-1.434548
C	-2.785463	0.940893	0.755662
H	-3.074712	0.172674	1.480622
H	-2.638102	1.872864	1.299229
H	-3.602826	1.061756	0.029713
C	1.438584	-4.329765	-1.575431
H	0.879309	-4.472894	-2.504156
H	2.493931	-4.438136	-1.815881
H	1.157584	-5.113597	-0.861865

TS_{FG}

Mo	1.366444	0.117604	-1.279551
P	-0.222240	1.543142	-0.113165
P	2.249983	-1.915186	-0.355124
N	-0.280492	-1.222606	-0.690048
N	-1.573220	0.514004	0.180570
N	1.127023	-3.014322	-1.092445
C	-1.515788	-0.745916	-0.350215
C	-2.673607	-1.517361	-0.520445
C	-2.529877	-2.828065	-0.940198
C	-1.272315	-3.383520	-1.139777
C	-0.160486	-2.556063	-0.991243
C	-0.012810	2.477202	1.469966
C	0.283971	1.521468	2.623308
C	-0.851392	2.733378	-1.415166
C	-1.779051	3.837920	-0.912920
C	1.603190	-3.574401	1.881983
C	3.085515	-1.592770	2.366582
C	3.933947	-2.526052	-0.842796
C	4.427737	-3.779684	-0.117003
C	2.549552	0.832498	-0.005137
O	3.343243	1.306077	0.727942
H	-3.657794	-1.063278	-0.417544
H	-3.415838	-3.438546	-1.092439
H	-1.149669	-4.430824	-1.380879
H	0.322132	2.087016	3.560955
H	1.249919	1.023479	2.494428
H	-0.488681	0.753550	2.728345
H	-2.155886	4.396687	-1.777517
H	-1.258716	4.549501	-0.264838
H	-2.646299	3.448673	-0.374259
H	1.321429	-3.576439	2.941329
H	2.444918	-4.261640	1.764800
H	0.753398	-3.965358	1.316160
H	2.732567	-1.583332	3.404118
H	3.375596	-0.573754	2.107888
H	3.978201	-2.223101	2.334277
H	5.336126	-4.128513	-0.620966
H	3.710796	-4.602744	-0.122735
H	4.692808	-3.566629	0.921529
C	4.967445	-1.402214	-0.738265
H	4.687218	-0.534938	-1.340794
H	5.118300	-1.079076	0.295826
H	5.926947	-1.776490	-1.111794
C	0.294395	3.293948	-2.258659
H	0.874902	2.498137	-2.739462
H	-0.116037	3.924604	-3.055583
H	0.987344	3.903312	-1.670486
C	1.025429	3.594575	1.356389
H	0.776814	4.316275	0.574399
H	1.056548	4.139723	2.306238
H	2.027731	3.207428	1.162941
C	3.635467	2.226437	-2.418558
O	4.712348	2.363322	-2.750508
H	1.079858	-1.515518	1.638095
C	1.965367	-2.144311	1.481487
H	3.799558	-2.750759	-1.908033
H	-1.437563	2.058710	-2.054521
H	-0.985949	2.950911	1.649595
Br	2.497038	-0.756052	-3.498580
Br	-5.715922	0.393261	-1.389537

C	-2.824431	0.941235	0.809213
H	-3.114663	0.205801	1.567218
H	-2.670688	1.895166	1.311380
H	-3.643908	1.035313	0.081357
C	1.377568	-4.368412	-1.552597
H	0.789005	-4.558945	-2.454582
H	2.426093	-4.479519	-1.823044
H	1.123751	-5.120821	-0.795800

G

Mo	1.310997	0.076312	-1.321818
P	-0.264926	1.526312	-0.150632
P	2.224271	-1.926341	-0.363911
N	-0.332727	-1.246259	-0.667573
N	-1.633746	0.517326	0.134139
N	1.087865	-3.032355	-1.074092
C	-1.568550	-0.766494	-0.333710
C	-2.719076	-1.560719	-0.440645
C	-2.566981	-2.885031	-0.809919
C	-1.307666	-3.430586	-1.027595
C	-0.203777	-2.585137	-0.938608
C	-0.035202	2.427173	1.450774
C	0.272339	1.439434	2.573690
C	-0.847252	2.748120	-1.441870
C	-1.787291	3.844121	-0.943530
C	1.637452	-3.544796	1.917938
C	3.105323	-1.525215	2.316111
C	3.899501	-2.551848	-0.866183
C	4.382060	-3.815278	-0.149185
C	2.563356	0.861135	-0.163184
O	3.392188	1.359893	0.512964
H	-3.707689	-1.118041	-0.330433
H	-3.447482	-3.513859	-0.909856
H	-1.178845	-4.483837	-1.237248
H	0.330260	1.979636	3.525107
H	1.233234	0.939824	2.414460
H	-0.503497	0.673903	2.671555
H	-2.121520	4.437514	-1.802409
H	-1.290478	4.526388	-0.247220
H	-2.679578	3.442771	-0.457565
H	1.373261	-3.526244	2.981567
H	2.484925	-4.224593	1.801186
H	0.782697	-3.957425	1.374868
H	2.789083	-1.506209	3.365177
H	3.357211	-0.502957	2.027870
H	4.013930	-2.130882	2.261350
H	5.279190	-4.175433	-0.665217
H	3.653610	-4.627815	-0.147428
H	4.663201	-3.607553	0.886396
C	4.952657	-1.445953	-0.760042
H	4.685747	-0.568401	-1.352253
H	5.118620	-1.137592	0.275893
H	5.901165	-1.836104	-1.145409
C	0.333199	3.329396	-2.224589
H	0.937992	2.551351	-2.706455
H	-0.047052	3.980746	-3.019836
H	0.999129	3.925256	-1.593656
C	1.007730	3.541840	1.363182
H	0.746709	4.300010	0.620914
H	1.063220	4.042928	2.336055
H	2.003114	3.158297	1.130669
C	4.062516	2.741473	-2.390897
O	5.159217	2.467316	-2.488277
H	1.081160	-1.500273	1.639416
C	1.971984	-2.119855	1.478248
H	3.757503	-2.770806	-1.931738
H	-1.411789	2.093594	-2.120717
H	-1.004557	2.898915	1.654351
Br	2.643189	-0.569932	-3.439585
Br	-5.813662	0.247465	-1.323734
C	-2.880925	0.961364	0.760243
H	-3.148041	0.269523	1.566586
H	-2.738555	1.947179	1.199908
H	-3.712752	0.998332	0.041437
C	1.333912	-4.393381	-1.518942
H	0.695239	-4.610515	-2.379598
H	2.365747	-4.491523	-1.852150
H	1.138712	-5.134403	-0.734238

H

Mo	1.267888	0.176593	-1.416637
P	-0.274856	1.640040	-0.216922
P	2.247240	-1.758709	-0.398388
N	-0.341585	-1.147374	-0.675151
N	-1.685465	0.645174	-0.033192
N	1.114543	-2.922877	-1.015758
C	-1.587776	-0.667119	-0.390967
C	-2.714146	-1.503827	-0.438428
C	-2.536839	-2.843054	-0.723428
C	-1.267645	-3.368256	-0.932792
C	-0.183601	-2.494610	-0.886218
C	-0.110956	2.415402	1.459484
C	0.053083	1.360633	2.548140
C	-0.781947	2.983010	-1.411334
C	-1.516807	4.194523	-0.838291
C	1.803960	-3.301046	1.974803
C	3.164199	-1.183153	2.233771
C	3.922512	-2.371921	-0.918366
C	4.458050	-3.584480	-0.153297
C	2.558324	1.043594	-0.362528
O	3.412190	1.596899	0.236243
H	-3.702645	-1.100645	-0.275840
H	-3.400923	-3.500305	-0.762470
H	-1.116379	-4.427146	-1.092138
H	0.046037	1.846599	3.530217
H	1.005955	0.831875	2.444889
H	-0.757473	0.625749	2.532937
H	-1.859185	4.812484	-1.676934
H	-0.870237	4.822169	-0.217201
H	-2.401147	3.934890	-0.252201
H	1.550186	-3.240409	3.039605
H	2.686406	-3.938420	1.884016
H	0.967461	-3.789750	1.467216
H	2.885222	-1.147813	3.292984
H	3.332572	-0.157136	1.900051
H	4.111901	-1.724605	2.164998
H	5.349110	-3.949320	-0.676442
H	3.751073	-4.413299	-0.086732
H	4.763101	-3.315695	0.861509
C	4.949859	-1.237270	-0.896050
H	4.649631	-0.402588	-1.532212
H	5.126512	-0.864416	0.116694
H	5.901012	-1.626222	-1.276338
C	0.421917	3.435456	-2.241912
H	0.869006	2.609424	-2.809430
H	0.091290	4.178855	-2.977081
H	1.209552	3.893199	-1.633817
C	0.999054	3.465329	1.522636
H	0.935174	4.206606	0.721607
H	0.921798	4.002929	2.474279
H	1.987602	3.004250	1.477597
C	4.040991	2.740272	-2.785200
O	5.130693	2.440910	-2.889273
H	1.126451	-1.312210	1.609238
C	2.049729	-1.884422	1.456113
H	3.759006	-2.645860	-1.967941
H	-1.461823	2.427579	-2.083908
H	-1.072007	2.925628	1.610648
Br	2.531200	-0.540304	-3.551799
Br	-2.756974	0.880212	-3.633120
C	-3.010686	1.206842	0.196736
H	-3.565650	0.600328	0.918387
H	-2.916880	2.203009	0.625067
H	-3.556087	1.267929	-0.753574
C	1.382289	-4.292764	-1.418160
H	0.736410	-4.552587	-2.261501
H	2.410912	-4.381260	-1.763380
H	1.215314	-5.010518	-0.605807

TS_{HI}

Mo	1.261219	0.180558	-1.420850
P	-0.279282	1.650951	-0.209021
P	2.239903	-1.752454	-0.404435
N	-0.344474	-1.137417	-0.679122
N	-1.688015	0.653356	-0.027643
N	1.108574	-2.914640	-1.025505
C	-1.589532	-0.659822	-0.385704
C	-2.715082	-1.497273	-0.424908
C	-2.540307	-2.834804	-0.719583
C	-1.272914	-3.357783	-0.941651
C	-0.188462	-2.484554	-0.894477
C	-0.121318	2.427651	1.466785
C	0.039499	1.370499	2.553842
C	-0.784319	2.989139	-1.412028
C	-1.534787	4.195273	-0.848333
C	1.795031	-3.302355	1.963206
C	3.147942	-1.180851	2.232948
C	3.916054	-2.360019	-0.926252
C	4.457269	-3.569551	-0.160654
C	2.545912	1.053425	-0.362727
O	3.397885	1.605961	0.239221
H	-3.702549	-1.096425	-0.250215
H	-3.404552	-3.491981	-0.755860
H	-1.121865	-4.415602	-1.108205
H	0.032121	1.854831	3.536674
H	0.991160	0.839514	2.450511
H	-0.772609	0.637401	2.536250
H	-1.860258	4.818571	-1.689699
H	-0.901630	4.819390	-0.210014
H	-2.430463	3.930416	-0.282337
H	1.537333	-3.245540	3.027280
H	2.679510	-3.937091	1.873518
H	0.961567	-3.791821	1.451384
H	2.865722	-1.151057	3.291497
H	3.312658	-0.152747	1.903832
H	4.098172	-1.717901	2.164636
H	5.351818	-3.928930	-0.681560
H	3.754772	-4.402431	-0.096676
H	4.757852	-3.299737	0.855239
C	4.937451	-1.220174	-0.907106
H	4.632340	-0.389378	-1.546172
H	5.111176	-0.842407	0.104413
H	5.891193	-1.604640	-1.285348
C	0.426724	3.450251	-2.228052
H	0.882520	2.627535	-2.793201
H	0.100712	4.194854	-2.963988
H	1.205067	3.909491	-1.609300
C	0.986918	3.478862	1.534412
H	0.920646	4.223198	0.736365
H	0.908397	4.012082	2.488382
H	1.976389	3.019862	1.487271
C	4.043094	2.727276	-2.782703
O	5.129982	2.416912	-2.883697
H	1.112997	-1.314433	1.601153
C	2.038568	-1.883456	1.449476
H	3.751792	-2.635608	-1.975331
H	-1.444335	2.422602	-2.093111
H	-1.083394	2.936873	1.615117
Br	2.454572	-0.555707	-3.566772
Br	-2.511145	0.681347	-3.734058
C	-3.013531	1.207969	0.213361
H	-3.555904	0.605948	0.948267
H	-2.923482	2.210148	0.627536
H	-3.577713	1.254531	-0.725934
C	1.375417	-4.283377	-1.431814
H	0.734932	-4.538033	-2.280896
H	2.406367	-4.373135	-1.769805
H	1.200893	-5.004042	-0.623624

I

Mo	1.256087	0.134756	-1.455452
P	-0.284834	1.644769	-0.398288
P	2.170768	-1.831280	-0.411275
N	-0.412588	-1.169900	-0.632715
N	-1.691428	0.683706	-0.048444
N	0.989879	-2.967014	-1.011687
C	-1.626628	-0.660219	-0.309170
C	-2.770405	-1.472483	-0.236301
C	-2.634895	-2.828980	-0.473272
C	-1.390371	-3.378333	-0.745032
C	-0.294495	-2.514695	-0.806131
C	-0.035052	2.533254	1.218396
C	0.295691	1.543942	2.333069
C	-0.861127	2.933336	-1.631524
C	-1.826326	4.000838	-1.116063
C	1.724591	-3.400814	1.943192
C	3.231859	-1.389862	2.200036
C	3.801908	-2.495554	-1.010612
C	4.300915	-3.773273	-0.328261
C	2.507905	0.936381	-0.269857
O	3.333590	1.470033	0.383546
H	-3.738648	-1.045971	-0.013844
H	-3.508616	-3.472705	-0.422500
H	-1.262298	-4.445278	-0.870367
H	0.330612	2.074525	3.291507
H	1.271807	1.075430	2.175717
H	-0.456850	0.753751	2.418302
H	-2.070324	4.675031	-1.945832
H	-1.388823	4.613160	-0.321477
H	-2.769031	3.586514	-0.753941
H	1.525733	-3.357145	3.020547
H	2.558802	-4.090529	1.791600
H	0.835211	-3.818586	1.462992
H	2.986762	-1.346499	3.267561
H	3.472154	-0.376113	1.873991
H	4.129786	-2.005540	2.097109
H	5.157838	-4.152130	-0.896789
H	3.560559	-4.572569	-0.273948
H	4.650163	-3.566031	0.686681
C	4.889474	-1.419189	-0.945904
H	4.612887	-0.525565	-1.509368
H	5.117952	-1.137119	0.085857
H	5.805906	-1.828758	-1.386149
C	0.325144	3.580114	-2.352132
H	1.017060	2.840255	-2.765622
H	-0.056716	4.183714	-3.184217
H	0.891741	4.246652	-1.695206
C	0.984792	3.669834	1.145530
H	0.675263	4.463254	0.461441
H	1.081624	4.118737	2.141095
H	1.973503	3.319576	0.843301
C	3.954449	3.797193	-1.527405
O	5.080868	3.694526	-1.624079
H	1.169161	-1.357268	1.651726
C	2.042443	-1.990218	1.449282
H	3.600033	-2.703754	-2.070523
H	-1.381157	2.298068	-2.360065
H	-1.010128	2.984797	1.442323
Br	3.006839	0.753638	-3.227294
Br	-0.353347	-0.047988	-3.554061
C	-2.933808	1.217582	0.492615
H	-3.266636	0.618013	1.346131
H	-2.779092	2.233758	0.848378
H	-3.728349	1.234646	-0.262402
C	1.207812	-4.339779	-1.430788
H	0.467629	-4.601762	-2.191624
H	2.188007	-4.430662	-1.897092
H	1.136468	-5.058217	-0.604504

J

Mo	0.039550	-0.514491	-0.094353
P	2.267496	0.128527	0.561473
P	-2.247421	0.153596	0.361194
N	0.012753	1.778053	-0.057855
N	2.304578	1.845092	0.432892
N	-2.322788	1.786063	-0.196233
C	1.166212	2.495049	0.009231
C	1.230183	3.856707	-0.317521
C	0.068337	4.497525	-0.705934
C	-1.132425	3.812808	-0.705372

C	-1.133431	2.460901	-0.331523
C	3.098286	-0.178878	2.200833
C	2.417776	0.661776	3.282802
C	3.377216	-0.498308	-0.837110
C	4.364531	0.548037	-1.350994
C	-3.898223	1.259392	2.461635
C	-3.335863	-1.191067	2.595934
C	-3.410617	-0.816015	-0.746171
C	-4.916062	-0.556646	-0.633427
C	-0.076201	-1.217492	1.660444
O	-0.183076	-1.726158	2.720110
H	2.171077	4.387378	-0.290108
H	0.096121	5.543923	-0.996313
H	-2.049959	4.313404	-0.978066
H	2.933761	0.503631	4.236398
H	1.373554	0.361837	3.416048
H	2.440410	1.733101	3.067228
H	4.879618	0.131718	-2.224384
H	5.128732	0.793508	-0.606935
H	3.870667	1.469312	-1.669072
H	-4.089828	1.227972	3.540677
H	-4.853074	1.091839	1.954602
H	-3.544609	2.265526	2.221971
H	-3.456197	-1.169067	3.684961
H	-2.625728	-1.986877	2.361996
H	-4.306064	-1.451109	2.163407
H	-5.433940	-1.295893	-1.255694
H	-5.216565	0.425684	-0.999899
H	-5.287900	-0.677037	0.387489
C	-3.146191	-2.322388	-0.640593
H	-2.086345	-2.578109	-0.718881
H	-3.532204	-2.738620	0.294487
H	-3.669922	-2.822128	-1.463645
C	4.096684	-1.810755	-0.544425
H	3.413105	-2.595842	-0.212882
H	4.569697	-2.158176	-1.470201
H	4.889779	-1.679514	0.198754
C	3.134136	-1.653341	2.610161
H	3.747966	-2.263173	1.948351
H	3.569267	-1.719238	3.614051
H	2.134659	-2.088979	2.652392
H	-1.943448	0.433688	2.666881
C	-2.865659	0.186243	2.124698
H	-3.055236	-0.486144	-1.731712
H	2.642945	-0.683938	-1.631860
H	4.135521	0.161810	2.081826
Br	-0.317586	-0.102210	-2.682405
Br	0.614866	-2.932375	-0.740587
C	3.468153	2.652320	0.782862
H	3.191680	3.420556	1.512710
H	4.233403	2.019425	1.228550
H	3.907318	3.138244	-0.093990
C	-3.557945	2.517578	-0.443093
H	-3.666316	2.753228	-1.508125
H	-4.408967	1.920034	-0.134501
H	-3.587235	3.447996	0.133088
